# Triphenylphosphine‐Catalyzed Synthesis of *β*‐Thiopropionate Thioesters or Esters via the Reaction of Maleic Anhydride with Thiols and Alcohols

**DOI:** 10.1002/open.202500089

**Published:** 2025-07-07

**Authors:** Hamed Ahmadi, Sepideh Abbasifard, Najmeh Nowrouzi, Mohammad Abbasi

**Affiliations:** ^1^ Department of Chemistry Faculty of Nano and Bio Science and Technology Persian Gulf University Bushehr 75169 Iran

**Keywords:** alcohols, maleic anhydrides, thiols, triphenylphosphines, *β* ‐thiopropionate thioesters

## Abstract

In this study, the one‐pot reaction of maleic anhydride with a combination of aromatic and aliphatic thiols, or with an alcohol and thiol mixture, in the presence of triphenylphosphine catalyst has been investigated. The reaction with the mixture of thiols occurs in a single step, and despite the presence of two different thiols in the reaction medium, a unique product is formed. The aromatic thiol facilitates the ring‐opening of the anhydride, while the aliphatic thiol participates solely in the Michael addition to the intermediate. When a mixture of alcohol and thiol is used in the medium, the reaction proceeds in two steps. In this reaction, the alcohol opens the anhydride ring, and the thiol induces nucleophilic addition to the acrylate intermediate.

## Graphical abstract

Decarboxylative ring‐opening of maleic anhydride with a combination of aromatic and aliphatic thiols or an alcohol/thiol mixture for the synthesis of *β*‐thiopropionate thioesters or esters, catalyzed by triphenylphosphine.

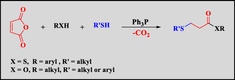

## 1 Introduction

The development of metal‐free methods for C—S bond formation has gained particular importance due to the essential role of organic sulfur compounds across multiple fields, especially in pharmaceuticals and materials science.^[^ ^1^ ^]^ Sulfur‐containing compounds are fundamental to many biologically active molecules, including antibiotics, antivirals, and anticancer agents.^[^ ^2^ ^]^ Additionally, they play a critical role in developing functional materials, such as conductive polymers, dyes, and catalysts.^[^ ^3^ ^]^ Metal‐free strategies offer significant advantages over metal‐catalyzed methods, including improved stability, cost‐effectiveness, and environmental safety. These methods also eliminate the risk of metal contamination, a critical factor for synthesizing biologically relevant compounds. Furthermore, metal‐free approaches often involve milder reaction conditions and simpler reagent handling, enhancing the overall efficiency and accessibility of the synthesis. This makes metal‐free C—S bond formation especially attractive in pharmaceutical applications, where avoiding metal contamination is essential, and in large‐scale industrial processes, where cost and environmental impact are key considerations.^[^ ^4^ ^]^

Thioesters are an important class of organosulfur compounds that play a vital role in biological chemistry and synthetic chemistry due to their unique reactivity that results from the presence of a sulfur atom instead of an oxygen atom in esters.^[^ ^5^ ^]^ In biochemistry, they are key intermediates in metabolic processes such as the citric acid cycle and fatty acid oxidation, of which acetyl‐CoA is a prime example.^[^ ^6^ ^]^ Thioesters are also valuable in organic synthesis, facilitating carbon–carbon bond formation, peptide synthesis, and various chemical transformations. In industry, they are used in the production of polymers, pharmaceuticals, and as stabilizers in plastics.^[^ ^7^ ^]^ Their versatility and reactivity make them indispensable in a wide range of applications. Given the significance of this class of compounds, a variety of synthetic methods have been developed and reported for their synthesis.^[^ ^8^ ^]^

**Scheme 1:**
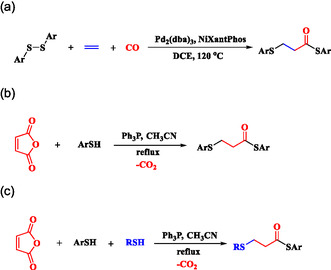
Synthesis of *β*‐thiopropionate thioesters. a) Wu's work. b) Our previous work. c) This work.

In 2021, Wu and coworkers reported a palladium‐catalyzed 1,2‐thiocarbonylation of ethylene using disulfides as a thiolating source, NiXantPhos as the ligand, and 1,2‐dichloroethane as the solvent, enabling the synthesis of *β*‐thiopropionate thioesters.^[^ ^9^ ^]^ This process involves the simultaneous introduction of two thiol molecules and a carbonyl group into ethylene, producing more complex and versatile C3 molecules, and requires a Pd‐NiXantphos catalyst system to facilitate the activation of CO and its incorporation into organic substrates (**Scheme** **1**a). Recently, we introduced a simple and metal‐free method for synthesizing *β*‐thiopropionate thioesters. In our study, the ring‐opening of maleic anhydride by thiols or disulfides, catalyzed by triphenylphosphine, facilitated the formation of two carbon–sulfur bonds.^[^ ^10^ ^]^ The first C–S bond was formed through the nucleophilic attack of a thiolate anion on maleic anhydride, while the second bond was established via a Michael addition of the second thiol molecule to an in situ generated acrylic thioester (Scheme 1b). In this system, aromatic thiols readily reacted with maleic anhydride, yielding thiopropionate thioester products (probably due to the greater acidity of the thiol proton in aromatic thiols), but aliphatic thiols such as benzyl mercaptan did not participate in the initial ring‐opening of maleic anhydride. Knowing that aliphatic thiols are effective in Michael addition reactions, we explored the simultaneous use of both aromatic and aliphatic thiols in the reaction medium. This approach allowed us to selectively introduce two distinct thiol groups into the target molecule (Scheme 1c).

## 2 Results and Discussion

In order to realize this idea, we decided to choose maleic anhydride (0.8 mmol), thiophenol (0.5 mmol) as aromatic thiol, benzyl mercaptan (0.5 mmol) as aliphatic thiol, and triphenylphosphine (0.25 mmol) as catalyst in refluxing CH_3_CN as the model substrates to establish this transformation. To our delight, 83% of the desired S‐phenyl 3‐(benzylthio)propanethioate was obtained from the model reaction (**Table** **1**, entry 1). Reducing the amount of triphenylphosphine and maleic anhydride reduced the yield, but increasing their amount did not affect the results (Table 1, entries 2, 3, and 5, 6). As expected, the reaction did not progress in the absence of triphenylphosphine (Table 1, entry 4). For solvent screening, EtOH, H_2_O, DMF, and toluene were used, but none were as effective as CH_*3*_CN. As shown in entries 7 to 10 of Table 1, in H_2_O, the desired product was not formed and the product yield in DMF and EtOH was lower than in CH_3_CN. The reaction in toluene resulted in a similar result with CH_3_CN, but CH_3_CN was chosen as the solvent due to milder reaction conditions. Finally, for temperature optimization, the model reaction was carried out at 40 °C and room temperature, both of which resulted in a decrease in yield (Table 1, entries 11 and 12).

**Table 1:** Optimization of the reaction conditions to produce *β*‐thiopropionate thioesters.

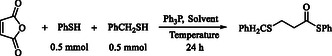					
Entry	Ph_3_P [mol%]	Maleic anhydride [mmol]	Solvent	Temperature	Yield [%]a)
1	50	0.8	CH_3_CN	reflux	83
2	40	0.8	CH_3_CN	reflux	74
3	60	0.8	CH_3_CN	reflux	84
4	–	0.8	CH_3_CN	reflux	–
5	50	0.7	CH_3_CN	reflux	73
6	50	0.9	CH_3_CN	reflux	83
7	50	0.8	EtOH	70	40
8	50	0.8	H_2_O	100	–
9	50	0.8	DMF	110	66
10	50	0.8	Toluene	reflux	82
11	50	0.8	CH_3_CN	40	56
12	50	0.8	CH_3_CN	r. t.	24

a) Isolated yields.

The optimized reaction conditions are as follows: maleic anhydride (0.8 mmol), aromatic thiol (0.5 mmol), and aliphatic thiol (0.5 mmol) in the presence of 0.25 mmol of triphenylphosphine, using CH_3_CN as the solvent under reflux conditions.

After achieving the optimal conditions, various aromatic and aliphatic thiols were investigated to assess the method's general applicability and scope for producing different derivatives of the product. The results of these investigations are presented in **Table** **2**.

**Table 2:** Substrate scope of *β*‐thiopropionate thioesters.

Entrya)	ArSH	RSH	Product	Yield [%]b)
1	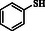	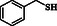	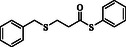	83
2	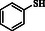	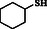	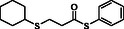	79
3	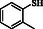	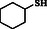	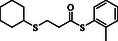	71
4	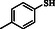	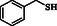	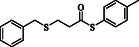	89
5	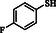	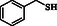	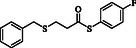	84
6	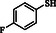	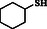	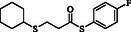	80
7	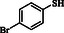	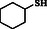	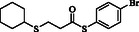	82
8	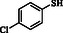	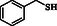	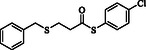	82
9	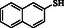	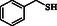	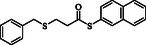	76
10	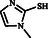	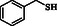	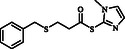	79
11	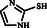	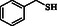	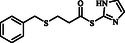	–
12	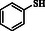	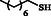	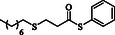	83
13	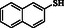	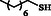	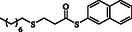	75
14	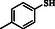	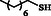	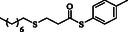	91
15	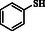	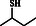	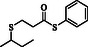	77
16	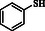	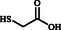	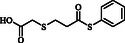	–

a) maleic anhydride (0.8 mmol), aromatic thiol (0.5 mmol), aliphatic thiol (0.5 mmol), Ph_3_P (0.25 mmol), CH_3_CN (1.0 mL), reflux, 24 h;

b) Isolated yield.

As the results indicate, the reported catalytic system is effective and selective, successfully avoiding the formation of mixed products despite the presence of two types of thiols in the reaction mixture. Thiophenol, 4‐methylthiophenol, 2‐methylthiophenol, 4‐fluorothiophenol, 4‐bromothiophenol, 4‐chlorothiophenol, naphthalene‐2‐thiol, and 1‐methyl‐1H‐imidazole‐2‐thiol were examined as aromatic thiols in this reaction. All of these thiols reacted effectively with maleic anhydride, producing the corresponding products in high yields. The results showed that the reaction is not very dependent on electronic effects, but is sensitive to steric effects. For example, the reaction with 2‐methylthiophenol demonstrates the negative impact of steric hindrance on reaction progress, resulting in a lower yield compared to 4‐methylthiophenol (Table 1, Entries 3 and 4). In contrast, 1H‐imidazole‐2‐thiol, which contains a free and unprotected amino group, failed to deliver the desired product under similar reaction conditions. This lack of reactivity may be attributed to the potential interference of the free nitrogen atom, which could compete as a nucleophile or interact with the catalyst, thereby disrupting the reaction pathway (Table 1, Entry 11).

Among the aliphatic thiols, benzyl mercaptan and 1‐octanethiol were used as primary aliphatic thiols, and cyclohexanethiol and 2‐butanethiol were employed as secondary ones. In all cases, the reactions proceeded effectively, yielding products with acceptable yields. In contrast, thioglycolic acid did not participate in the reaction, possibly due to its acidic functional group, which may suppress the desired transformation (Table 1, Entry 16).

**Scheme 2:**
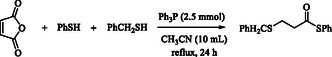
Gram scale reaction.

To demonstrate the practical utility and scalability of the reaction, a Gram scale synthesis was performed using maleic anhydride (0.78 g, 8.0 mmol), thiophenol (0.51 mL, 5.0 mmol), and benzyl mercaptan (0.59 mL, 5.0 mmol) under the optimized conditions. As shown in **Scheme** **2**, S‐phenyl 3‐(benzylthio)propanethioate was obtained in 78% isolated yield, affording 1.12 g of the desired product (Scheme 2).

After successfully accomplishing the Ph_3_P‐catalyzed decarboxylative ring‐opening of maleic anhydride with thiols, we decided to investigate the synthesis of *β*‐thiopropionate esters by using a mixture of alcohol and thiol in the reaction medium.

To achieve this goal, the reaction of maleic anhydride, benzyl alcohol, and thiophenol was selected as a model reaction and entered into the reaction in the optimal conditions obtained for the synthesis of *β*‐thiopropionate thioesters. For this purpose, maleic anhydride (0.8 mmol), benzyl alcohol (0.5 mmol), thiophenol (0.5 mmol), and triphenylphosphine (0.25 mmol) were added to the flask containing CH_3_CN (1 mL) and the mixture was allowed to mix for 24 h in reflux conditions. Investigating the progress of the reaction by thin layer chromatography (TLC) did not show a specific product up to 24 h. Then the reaction was repeated in toluene solvent and under reflux conditions, which again did not lead to the desired result. Following the failure to perform the reaction in one step, we decided to perform the reaction in two steps. First, maleic anhydride (0.8 mmol) and benzyl alcohol (0.5 mmol) were reacted in the presence of triphenylphosphine (50 mol%) in refluxing toluene. After 24 h, thiophenol (0.5 mmol) was added to the reaction mixture, followed by further refluxing for another 24 h. TLC analysis confirmed product formation, which was isolated and purified. Nuclear magnetic resonance (NMR) analysis confirmed the structure of the product as benzyl 3‐(phenylthio)propanoate in 50% yield (**Table** **3**, entry 1), indicating successful ring‐opening of maleic anhydride by the alcohol and subsequent Michael addition of thiophenol. Reducing the amount of triphenylphosphine up to 10 mol% did not have a significant effect on the results (Table 3, entries 2 and 3), but further reductions led to a decrease in yield (Table 3, entry 4). Next, the effect of various solvents such as H_2_O, EtOH, CH_3_CN, and DMF on the reaction system was studied (Table 3, entries 5–8). Among these solvents, refluxing in toluene provided the best results. Consequently, toluene was selected as the solvent. Performing the reaction at lower temperatures (80 °C and room temperature) showed that reducing the temperature led to a decrease in yield (Table 3, entries 9 and 10). In the next attempt, during the second step of the reaction, K_2_CO_3_ (0.5 mmol) was added as a base along with the thiol, which significantly increased the yield (Table 3, entry 11). Increasing the amount of K_2_CO_3_ or replacing it with bases such as NaOAc, NaOH, and Bu_3_N did not yield better results (Table 3, entries 12–15). Therefore, K_2_CO_3_ was chosen as the optimal base. After confirming the effective role of the base in the system, the model reaction was repeated in a single step in the presence of K_2_CO_3_, but the desired product was not formed (Table 3, entry 16). In another attempt, the reaction was carried out in the presence of K_2_CO_3_ and in the absence of triphenylphosphine, under which conditions the desired product was not formed. This result highlights the importance of triphenylphosphine in this protocol (Table 3, entry 17).

**Table 3:** Optimization of the reaction conditions for the synthesis of *β*‐thiopropionate esters.

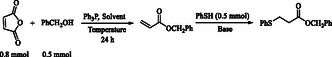					
Entry	Ph_3_P [mol%]	Solvent	Temperature	Base	Yield [%]a)
1	50	Toluene	reflux	–	50
2	30	Toluene	reflux	–	48
3	10	Toluene	reflux	–	48
4	8	Toluene	reflux	–	32
5	10	H_2_O	100	–	10
6	10	EtOH	70	–	18
7	10	CH_3_CN	reflux	–	35
8	10	DMF	110	–	22
9	10	Toluene	80	–	21
10	10	Toluene	r. t.	–	trace
11	10	Toluene	reflux	K_2_CO_3_ (0.5)	86
12	10	Toluene	reflux	K_2_CO_3_ (1.0)	86
13	10	Toluene	reflux	NaOAc (0.5)	64
14	10	Toluene	reflux	NaOH (0.5)	68
15	10	Toluene	reflux	Bu_3_N (0.5)	57
16b)	10	Toluene	reflux	K_2_CO_3_ (0.5)	–
17	–	Toluene	reflux	K_2_CO_3_ (0.5)	–

a) Isolated yields;

b) The reaction was carried out in one step.

Finally, the optimized conditions were obtained as follows: Step 1: Reaction of 0.5 mmol alcohol, 0.8 mmol maleic anhydride, and 10 mol% triphenylphosphine under reflux in toluene for 24 h.; Step 2: Addition of 0.5 mmol thiol and 0.5 mmol potassium carbonate, followed by further reflux in toluene for 24 h.

After confirming the structure of the desired product and obtaining the most suitable and optimal conditions, the reaction between maleic anhydride with various alcohols and thiols was carried out to produce *β*‐thiopropionate ester derivatives, the results of which are given in **Table** **4**.

**Table 4:** Substrate scope of *β*‐thiopropionate esters.

Entrya)	ROH	RSH	Product	Yield [%]b) ^,ref^
1	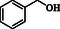	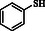	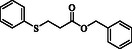	86^[^ ^13^ ^]^
2	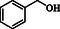	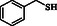	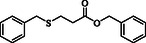	88
3	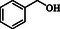	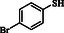	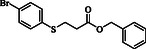	81^[^ ^13^ ^]^
4	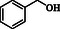	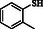	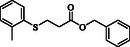	68^[^ ^13^ ^]^
5	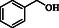	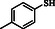	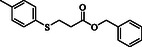	89^[^ ^13^ ^]^
6	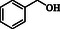	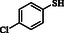	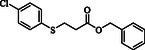	79^[^ ^13^ ^]^
7	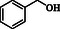	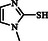	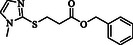	75
8	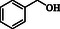	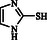	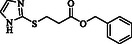	–
9	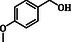	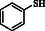	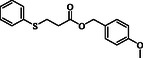	87
10	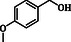	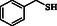	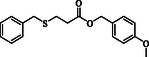	94
11	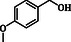	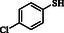	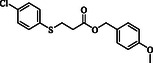	82
12	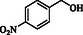	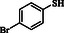	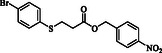	70
13	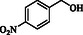	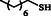	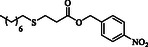	76
14	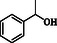	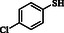	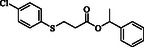	68
15	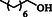	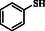	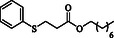	79^[^ ^14^ ^]^
16	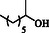	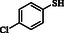	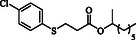	70
17	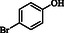	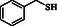	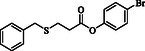	–
18	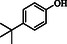	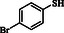	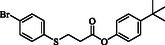	–

a) Reaction conditions: Step 1: 0.5 mmol alcohol, 0.8 mmol maleic anhydride, and 10 mol% triphenylphosphine under reflux in toluene for 24 h; Step 2: 0.5 mmol thiol and 0.5 mmol potassium carbonate, followed by further reflux in toluene for 24 h;

b) Isolated yield.

The results obtained have been satisfactory, and the good yields of the products indicate a favorable progress of the reactions. The reactions of benzyl alcohols with different thiols show that electron‐donating groups on the benzyl ring increase the product yield, while electron‐withdrawing groups somewhat decrease the yield. The results obtained in entries 6, 14, and 16 of Table 4 show that primary alcohols are more effective in these reactions compared to secondary ones. For instance, the reaction of benzyl alcohol, 1‐phenylethanol, and 2‐octanol with 4‐chlorothiophenol produced the corresponding products with yields of 79%, 68%, and 70%, respectively. In the latter two cases, where secondary alcohols were used, the yield decreased. Overall, benzyl alcohols perform better than linear alcohols in the studied system (compare entries 1 and 15 of Table 4). In another attempt, phenols were used instead of alcohols, but no product was formed (Table 4, entries 17 and 18). Increasing the amount of triphenylphosphine and base also did not help in forming the product with phenols. The inactivity of phenols is likely due to the reduced nucleophilicity of the phenolic oxygen because of resonance with the aromatic ring.

To examine the scope of the method, thiophenol and its derivatives (4‐bromothiophenol, 4‐chlorothiophenol, 4‐methylthiophenol, and 2‐methylthiophenol) were used in reactions with various alcohols. The results show that these reactions are influenced by both electronic and steric factors. Electron‐withdrawing bromine and chlorine groups on the aryl ring (Table 4, entries 1, 3, and 6) and a methyl group at the ortho position of the thiol group reduced the product yield (Table 4, entries 1 and 4). Benzyl mercaptan and 1‐octanethiol, as primary thiols, performed well in the studied system and produced the desired ester products with good to excellent yields when reacting with alcohols and maleic anhydride (Table 4, entries 2, 10, 13). 1‐Methyl‐1H‐imidazole‐2‐thiol was also used as another thiol derivative in the reaction with benzyl alcohol, producing the product with a 75% yield (Table 4, entry 7). However, 1H‐imidazole‐2‐thiol did not give satisfactory results, as it failed to produce the desired product in its reaction with benzyl alcohol (Table 4, entry 8). In this case, monitoring the progress of the reaction by TLC showed the formation of several unknown products. This can be due to the presence of a free nitrogen atom in this molecule, which can act as a nucleophile.

**Scheme 3:**
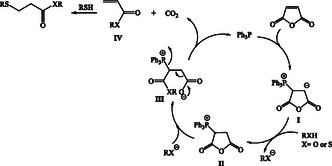
Proposed mechanism.

Based on the obtained results and previous reports,^[^ ^11^ ^]^ the following mechanism is proposed for the synthesis of *β*‐thiopropionate thioesters/esters (**Scheme** **3**). Initially, the addition of triphenylphosphine to maleic anhydride forms intermediate **I**, which then reacts with an alcohol or thiol to produce the phosphonium salt **II**. At this step, the ring is opened by the nucleophilic attack of an alkoxide or thiolate anion, generating intermediate **III**. The elimination of triphenylphosphine and carbon dioxide from this intermediate produces acrylate **IV**, which subsequently undergoes nucleophilic attack by a thiol, yielding the final product.

## 3 Conclusion

In this study, various *β*‐thiopropionate thioesters/ester derivatives were synthesized using a stable triphenylphosphine catalyst in a one‐pot process. The starting materials used are readily available and inexpensive. Maleic anhydride is a colorless solid that is widely produced on an industrial scale for use in coatings and polymers. Its chemistry is highly versatile due to its availability and high reactivity.^[^ ^12^ ^]^ Triphenylphosphine is also an inexpensive compound, existing as relatively stable, colorless crystals at room temperature. Its nucleophilic and reducing properties make it a valuable reagent in organic synthesis and an effective ligand for organometallic complexes. Alcohols and thiols are also readily available and relatively inexpensive compounds. The high‐to‐excellent yields, simplicity of the procedure, one‐pot nature of the reaction, inexpensive catalyst, and easy access to starting materials are additional advantages of this method.

## Conflict of Interest

The authors declare no conflicts of interest.

## Supporting information

Supplementary Material

## Data Availability

The data that support the findings of this study are available in the supplementary material of this article.
