# Supplementary material for: Triphenylphosphine‐Catalyzed Synthesis of β‐Thiopropionate Thioesters or Esters via the Reaction of Maleic Anhydride with Thiols and Alcohols
Source: ChemistryOpen. 2025 Jul 7;14(11):e202500089. doi: 10.1002/open.202500089 (PMC12598836; doi:10.1002/open.202500089)
Supplement: Supplementary file 1 — Supplementary Material [file OPEN-14-e202500089-s001.pdf]

Supporting Information

Triphenylphosphine-Catalyzed Synthesis of *β*-Thiopropionate Thioesters or Esters *via* the Reaction of Maleic Anhydride with Thiols and Alcohols

Hamed Ahmadi,[a] Sepideh Abbasifard,[a] Najmeh Nowrouzi*[a] and Mohammad Abbasi[a]

[a]Department of Chemistry, Faculty of Nano and Bio Science and Technology, Persian Gulf University, Bushehr 75169 Iran

E-mail address: nowrouzi@pgu.ac

General Information

All reagents and solvents were obtained from commercial suppliers and were purified by standard methods prior to use. Column chromatography was performed on silica gel using small columns. The progress of the reactions was monitored by thin-layer chromatography (TLC) using UV light at 254 nm. ^{1}H NMR (300 and 400 MHz) and ^{13}C NMR (75 and 100 MHz) spectra were recorded on a Bruker Avance DRX spectrometer in pure deuterated chloroform ($CDCl_3$). Chemical shifts (δ) are reported in parts per million (ppm) relative to tetramethylsilane (TMS) as an internal standard. Coupling constants (J) are given in hertz (Hz). Data for ^{1}H NMR are reported as follows: chemical shift (δ ppm), multiplicity (s = singlet, d = doublet, t = triplet, q = quartet, m = multiplet, dd = doublet of doublets, ddd = doublet of doublet of doublets, td = triplet of doublets), coupling constant (Hz), and integration.

General Procedure for the Synthesis of *β*-Thiopropionate Thioesters:

In a round-bottom flask, maleic anhydride (0.8 mmol), triphenylphosphine (0.25 mmol), aliphatic thiol (0.5 mmol), aromatic thiol (0.5 mmol), and CH_3CN (1 mL) were added. The reaction mixture was stirred in an oil bath under reflux conditions at 80 °C. The progress of the reaction was monitored by thin-layer chromatography (TLC). After 24 hours, and upon completion of the reaction, the mixture was allowed to cool to room temperature. The organic phase containing the product was purified by column chromatography on silica gel using *n*-hexane/ethyl acetate (10:1) as the eluent to afford the pure product.

General Procedure for the Synthesis of *β*-Thiopropionate Esters:

To a round-bottom flask containing toluene (2 mL) were added maleic anhydride (0.8 mmol), alcohol (0.5 mmol), and triphenylphosphine (0.05 mmol). The mixture was stirred under reflux conditions at 110 °C using a magnetic stirrer. The progress of the reaction was monitored by thinlayer chromatography (TLC). After 24 hours, thiol (0.5 mmol) and potassium carbonate (0.5 mmol) were added to the reaction mixture, and the reaction progress was again monitored by TLC. Upon completion (24 h), the mixture was allowed to cool to room temperature. Distilled water (1 mL) and ethyl acetate (3 × 1 mL) were added, and the organic layer was separated. The crude product was then purified by column chromatography on silica gel using *n*-hexane/ethyl acetate (10:1) as the eluent to afford the pure product.

S-phenyl 3-(benzylthio)propanethioate

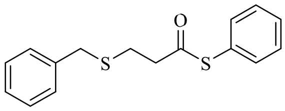

Yellow liquid; Yield: 83% (0.120 g), ^{1}H-NMR (300 MHz, $CDCl_3$), δ (ppm): 2.90 (t, J = 7.2 Hz, 2H, CH_2), 3.25 (t, J = 7.2 Hz, 2H, CH_2), 4.18 (s, 2H, CH_2), 7.23-7.42 (m, 10H, Ar). ^{13}C-NMR (75 MHz, $CDCl_3$) δ (ppm): 29.2, 33.3, 43.3, 126.7, 127.3, 128.7, 128.8, 129.1, 130.2, 134.9, 137.2, 196.8.

Anal. Calc. for $C_{16}H_{16}OS_2$: C, 66.63; H, 5.59; O, 5.55; S, 22.23. Found: C, 66.51; H, 5.50; S, 22.34.

S-phenyl 3-(cyclohexylthio)propanethioate

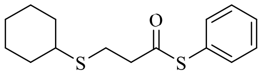

Yellow liquid; Yield: 79% (0.110 g), ^{1}H-NMR (300 MHz, $CDCl_3$), δ (ppm): 0.90-0.98 (m, 4H), 1.36-1.50 (m, 4H), 1.63-1.74 (m, 2H), 2.99 (t, J = 6.0 Hz, 2H, CH_2), 3.27 (t, J = 6.0 Hz, 2H, CH_2), 4.22-4.29 (m, 1H, CH), 7.24-7.42 (m, 5H, Ar). ^{13}C-NMR (75 MHz, $CDCl_3$) δ (ppm): 24.5, 28.9, 29.2, 30.3, 38.7, 43.2, 128.8, 129.1, 129.5, 130.2, 195.7.

Anal. Calc. for $C_{15}H_{20}OS_2$: C, 64.24; H, 7.19; O, 5.70; S, 22.86. Found: C, 64.40; H, 7.11; S, 22.80.

S-o-tolyl 3-(cyclohexylthio)propanethioate

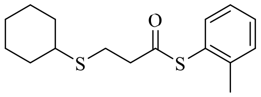

Yellow liquid; Yield: 71% (0.105 g), ^{1}H-NMR (300 MHz, $CDCl_3$), δ (ppm): 0.76-0.87 (m, 4H, $2CH_2$), 1.21-1.26 (m, 6H, $3CH_2$), 2.10 (s, 3H, CH_3), 2.62 (t, J = 6.0 Hz, 2H, CH_2), 3.08 (t, J = 6.0 Hz, 2H, CH_2), 4.09-4.16 (m, 1H, CH), 7.02-7.11 (m, 3H, Ar), 7.24 (t, J = 6.0 Hz, 1H, Ar).^{13}C-NMR (75 MHz, $CDCl_3$) δ (ppm): 23.0, 23.7, 24.5, 28.9, 30.4, 38.7, 43.2, 126.7, 127.2, 128.8, 130.2, 132.4, 134.5, 195.7.

Anal. Calc. for $C_{16}H_{22}OS_2$: C, 65.26; H, 7.53; O, 5.43; S, 21.77. Found: C, 65.32; H, 7.61; S, 21.70.

S-p-tolyl 3-(benzylthio)propanethioate

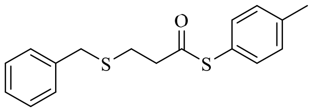

Yellow liquid; Yield: 89% (0.134 g), ^{1}H-NMR (300 MHz, $CDCl_3$), δ (ppm): 2.24 (s, 3H, CH_3), 2.75 (t, J = 6.0 Hz, 2H, CH_2), 3.07 (t, J = 6.0 Hz, 2H, CH_2), 4.05 (s, 2H, CH_2), 7.02-7.04 (m, 2H, Ar), 7.14-7.25 (m, 7H, Ar).^{13}C-NMR (75 MHz, $CDCl_3$) δ (ppm): 21.0, 30.0, 33.3, 43.4, 127.3, 128.6, 128.8, 129.9, 131.1, 131.1, 137.0, 137.3, 196.9.

Anal. Calc. for $C_{17}H_{18}OS_2$: C, 67.51; H, 6.00; O, 5.29; S, 21.20. Found: C, 67.59; H, 6.03; S, 21.16.

S-(4-fluorophenyl) 3-(benzylthio)propanethioate

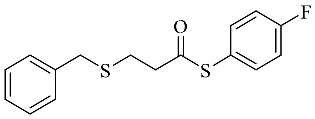

Yellow liquid; Yield: 84% (0.128 g), ^{1}H-NMR (300 MHz, $CDCl_3$), δ (ppm): 2.74 (t, J = 9.0 Hz, 2H, CH_2), 3.07 (t, J = 9.0 Hz, 2H, CH_2), 4.06 (s, 2H, CH_2), 6.89-6.97 (m, 2H, Ar), 7.13-7.34 (m, 7H, Ar).^{13}C-NMR (75 MHz, $CDCl_3$) δ (ppm): 30.6, 33.3, 43.2, 116.1, 116.4, 127.4, 128.7, 128.8, 133.4, 133.5, 137.2, 196.7.

Anal. Calc. for $C_{16}H_{15}FOS_2$: C, 62.72; H, 4.93; F, 6.20; O, 5.22; S, 20.93. Found: C, 62.62; H, 5.03; S, 21.00.

S-(4-fluorophenyl) 3-(cyclohexylthio)propanethioate

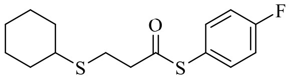

Yellow liquid; Yield: 80% (0.119 g),^{1}H-NMR (300 MHz, $CDCl_3$), δ (ppm): 1.40-1.49 (m, 4H), 1.59-1.77 (m, 4H), 1.90-1.97 (m, 2H), 2.80 (t, J = 6.0 Hz, 2H, CH_2), 3.15 (t, J = 6.0 Hz, 2H, CH_2), 3.51-3.61 (m, 1H, CH), 7.05 (t, J = 9.0 Hz, 2H, Ar), 7.40-7.44 (m, 2H, Ar). ^{13}C-NMR (75 MHz, $CDCl_3$) δ (ppm): 25.5, 25.9, 30.6, 32.9, 42.5, 43.6, 116.2 (d, J_{C-F} = 22.5 Hz), 120.7, 129.9, 133.3 (d, J_{C-F} = 8.25 Hz), 197.2.

Anal. Calc. for $C_{15}H_{19}FOS_2$: C, 60.37; H, 6.42; F, 6.37; O, 5.36; S, 21.49. Found: C, 60.45; H, 6.35; S, 21.60.

_S_-(4-bromophenyl) 3-(cyclohexylthio)propanethioate

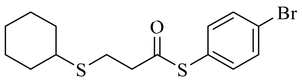

Yellow liquid; Yield: 82% (0.146 g),^{1}H-NMR (300 MHz, $CDCl_3$), δ (ppm): 1.29-1.49 (m, 4H), 1.58-1.77 (m, 4H), 1.90-1.97 (m, 2H), 2.83 (t, J = 6.0 Hz, 2H, CH_2), 3.20 (t, J = 6.0 Hz, 2H, CH_2), 3.53-3.61 (m, 1H, CH), 7.24-7.31 (m, 2H, Ar), 7.43-7.48 (m, 2H, Ar).^{13}C-NMR (75 MHz, $CDCl_3$) δ (ppm): 25.1, 25.9, 29.3, 32.9, 42.6, 43.4, 120.5, 131.6, 132.1, 134.4, 197.1.

Anal. Calc. for $C_{15}H_{19}BrOS_2$: C, 50.14; H, 5.33; Br, 22.24; O, 4.45; S, 17.84. Found: C, 50.21; H, 5.40; S, 17.80.

S-(4-chlorophenyl) 3-(benzylthio)propanethioate

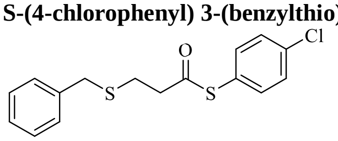

Yellow liquid; Yield: 82% (0.132 g),^{1}H-NMR (400 MHz, DMSO-d_6), δ (ppm): 2.92 (t, J = 8.0 Hz, 2H, CH_2), 3.03 (t, J = 8.0 Hz, 2H, CH_2), 4.15 (s, 2H, CH_2), 7.30-7.32 (m, 2H, Ar), 7.38-7.43 (m, 6H, Ar), 7.537.56 (m, 1H, Ar).^{13}C-NMR (100 MHz, DMSO-d_6) δ (ppm): 28.3, 32.7, 42.8, 127.6, 129.0, 129.2, 129.5, 129.9, 130.7, 130.9, 136.5, 196.9.

Anal. Calc. for $C_{16}H_{15}ClOS_2$: C, 59.52; H, 4.68; Cl, 10.98; O, 4.96; S, 19.86. Found: C, 59.61; H, 4.74; S, 19.75.

_S_-naphthalen-2-yl 3-(benzylthio)propanethioate

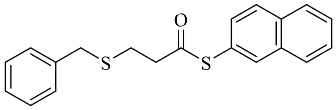

Kream Solid; Yield: 76% (0.128 g),^{1}H-NMR (300 MHz, $CDCl_3$), δ (ppm): 2.82 (t, J = 7.8 Hz, 2H, CH_2), 3.22 (t, J = 7.2 Hz, 2H, CH_2), 4.05 (s, 2H, CH_2), 7.15-7.23 (m, 5H, Ar), 7.33-7.43 (m, 3H, Ar), 7.66-7.73 (m, 4H, Ar).^{13}C-NMR (75 MHz, $CDCl_3$) δ (ppm): 29.1, 33.3, 43.2, 126.0, 126.9, 127.2, 127.3, 127.7, 127.8, 128.2, 128.7, 128.7, 128.8, 132.0, 132.3, 133.7, 137.2, 196.8.

Anal. Calc. for $C_{20}H_{18}OS_2$: C, 70.97; H, 5.36; O, 4.73; S, 18.94. Found: C, 70.88; H, 5.41; S, 19.01.

S-(1-methyl-1*H*-imidazol-2-yl) 3-(benzylthio)propanethioate

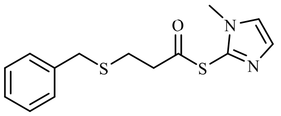

Yellow liquid; Yield: 79% (0.115 g), ^{1}H-NMR (300 MHz, $CDCl_3$) δ (ppm): 2.99-3.02 (m, 2H, CH_2), 3.033.06 (m, 2H, CH_2), 3.68 (s, 2H, CH_2), 3.79 (s, 3H, CH_3), 7.11-7.12 (m, 1H, Ar), 7.18 (d, J = 2.0 Hz, 1H, Ar), 7.24-7.30 (m, 1H, Ar), 7.33-7.37 (m, 4H, Ar). ^{13}C-NMR (75 MHz, $CDCl_3$) δ (ppm): 28.9, 34.2, 37.7, 41.0, 124.0, 128.4, 129.6, 129.8. 129.9, 135.2, 139.1, 192.1.

Anal. Calc. for $C_{14}H_{16}N_2OS_2$: C, 57.51; H, 5.52; N, 9.58; O, 5.47; S, 21.93. Found: C, 57.42; H, 5.50; N, 9.64; S, 21.99.

***S*-phenyl 3-(octylthio)propanethioate**

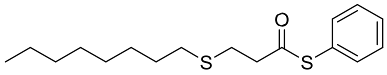

Yellow liquid; Yield: 83% (0.128 g),^{1}H-NMR (300 MHz, $CDCl_3$), δ (ppm): 0.89-0.98 (m, 9H), 1.42-1.50 (m, 2H, CH_2), 1.54-1.64 (m, 3H, CH_2), 1.68-1.76 (m, 1H, CH_2), 2.84-2.94 (m, 4H, $2CH_2$), 3.22 (t, J = 7.2 Hz, 2H, CH_2), 7.22-7.42 (m, 5H, Ar).^{13}C-NMR (75 MHz, $CDCl_3$) δ (ppm): 10.9, 14.1, 22.6, 23.0, 23.7, 29.4, 30.3, 31.8, 38.7, 43.5, 126.6, 128.8, 129.0, 130.0, 197.7.

Anal. Calc. for $C_{17}H_{26}OS_2$: C, 65.76; H, 8.44; O, 5.15; S, 20.65. Found: C, 65.75; H, 8.51; S, 20.69.

***S*-naphthalen-2-yl 3-(octylthio)propanethioate**

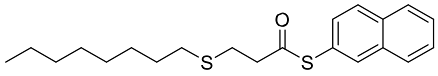

Yellow liquid; Yield: 75% (0.135 g),^{1}H-NMR (300 MHz, $CDCl_3$), δ (ppm): 0.77-0.86 (m, 6H), 1.24,1.36 (m, 6H), 1.42-1.51 (m, 3H), 2.77-2.82 (m, 4H), 3.21 (t, J = 7.2 Hz, 2H), 7.34-7.48 (m, 3H, Ar), 7.61-7.73 (m, 4H, Ar). ^{13}C-NMR (75 MHz, $CDCl_3$) δ (ppm): 10.9, 14.1, 22.6, 23.7, 28.8, 29.0, 30.3, 31.8, 38.7, 43.5, 125.9, 126.6, 127.2, 127.8, 128.1, 128.6, 130.9, 132.0, 132.5, 133.7, 197.7.

Anal. Calc. for $C_{21}H_{28}OS_2$: C, 69.95; H, 7.83; O, 4.44; S, 17.78. Found: C, 69.88; H, 7.90; S, 17.70.

***S*-*p*-tolyl 3-(octylthio)propanethioate**

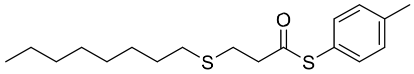

Yellow liquid; Yield: 91% (0.147 g),^{1}H-NMR (300 MHz, $CDCl_3$), δ (ppm): 0.90 (t, J = 6.6 Hz, 3H), 1.261.38 (m, 9H), 1.57-1.65 (m, 3H), 2.25 (t, J = 3.6 Hz, 2H), 2.29 (s, 3H, CH_3), 2.82 (t, J = 7.8 Hz, 2H,

CH_2), 3.09 (t, *J* = 6.9 Hz, 2H, CH_2), 7.42-7.48 (m, 2H, Ar), 7.60-7.66 (m, 2H, Ar). ^{13}C-NMR (75 MHz, $CDCl_3$) δ (ppm): 10.9, 14.0, 21.0, 21.3, 23.0, 23.7, 28.9, 29.9, 30.3, 38.7, 43.2, 130.9, 131.1, 134.4, 139.8, 196.3.

Anal. Calc. for $C_{18}H_{28}OS_2$: C, 66.62; H, 8.70; O, 4.93; S, 19.76. Found: C, 66.53; H, 8.77; S, 19.86.

_S_-phenyl 3-(sec-butylthio)propanethioate

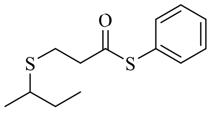

Yellow liquid; Yield: 77% (0.098 g),^{1}H-NMR (300 MHz, $CDCl_3$), δ (ppm): 0.96 (t, *J* = 7.5 Hz, 3H, CH_3), 1.33-1.47 (m, 4H), 1.68-1.76 (m, 1H), 2.99 (t, *J* = 7.8 Hz, 2H, CH_2), 3.27 (t, *J* = 6.9 Hz, 2H, CH_2), 4.244.27 (m, 1H, CH), 7.25-7.38 (m, 3H, Ar), 7.54-7.58 (m, 1H, Ar), 7.72-7.75 (m, 1H, Ar). ^{13}C-NMR (75 MHz, $CDCl_3$) δ (ppm): 14.0, 23.0, 28.9, 29.2, 38.7, 43.2, 129.1, 129.2, 130.2, 134.5, 195.7.

Anal. Calc. for $C_{13}H_{18}OS_2$: C, 61.38; H, 7.13; O, 6.29; S, 25.20. Found: C, 61.44; H, 7.19; S, 25.14.

benzyl 3-(phenylthio)propionoate

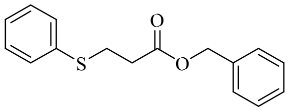

Oily liquid; Yield: 86% (0.117 g),^{1}H-NMR (300 MHz, $CDCl_3$) δ (ppm): 2.72 (t, *J* = 7.5 Hz, 2H, CH_2), 3.22 (t, *J* = 7.5 Hz, 2H, CH_2), 5.16 (s, 2H, CH_2), 7.22-7.44 (m, 10H, Ar). ^{13}C-NMR (75 MHz, $CDCl_3$) δ (ppm):
29.1, 34.4, 66.6, 126.6, 128.3, 128.3, 128.6, 129.0, 130.2, 135.1, 135.7, 171.6.

Deleted: ¶

benzyl 3-(benzylthio)propanoate

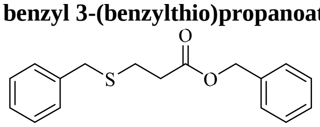

Oily liquid; Yield: 88% (0.126 g),^{1}H-NMR (300 MHz, $CDCl_3$) δ (ppm): 2.53 (t, *J* = 6.0 Hz, 2H, CH_2), 2.63 (t, *J* = 6.0 Hz, 2H, CH_2), 3.65 (s, 2H, CH_2), 5.05 (s, 2H, CH_2), 7.19-7.32 (m, 10H, Ar). ^{13}C-NMR (75 MHz, $CDCl_3$) δ (ppm): 26.2, 34.5, 36.3, 66.5, 127.1, 128.2, 128.3, 128.5, 128.6, 128.8, 135.7, 138.0, 171.7.

Anal. Calc. for $C_{17}H_{18}O_2S$: C, 71.30; H, 6.34; O, 11.17; S, 11.19. Found: C, 71.39; H, 6.40; S, 11.11.

benzyl 3-((4-bromophenyl)thio)propanoate

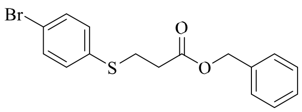

Light yellow liquid; Yield: 81% (0.142 g),^{1}H-NMR (300 MHz, $CDCl_3$) δ (ppm): 2.58 (t, J = 6.0 Hz, 2H, CH_2), 3.09 (t, J = 6.0 Hz, 2H, CH_2), 5.05 (s, 2H, CH_2), 7.15-7.19 (m, 2H, Ar), 7.24-7.36 (m, 6H, Ar), 7.447.65 (m, 1H, Ar). ^{13}C-NMR (75 MHz, $CDCl_3$) δ (ppm): 29.1, 34.2, 66.6, 120.6, 128.3, 128.4, 128.6, 131.7, 132.1, 134.3, 135.6, 171.4.

benzyl 3-(*o*-tolylthio)propanoate

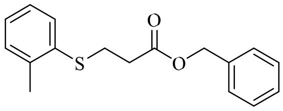

Light brown liquid; Yield: 68% (0.097 g),^{1}H-NMR (300 MHz, $CDCl_3$) δ (ppm): 2.40 (s, 3H, CH_3), 2.72 (t, J = 7.5 Hz, 2H, CH_2), 3.20 (t, J = 7.5 Hz, 2H, CH_2), 5.16 (s, 2H, CH_2), 7.31-7.46 (m, 7H, Ar), 7.56 (dd, J_1 = 3.5, J_2 = 2.5 Hz, 1H, Ar), 7.74 (dd, J_1 = 3.5, J_2 = 2.5 Hz, 1H, Ar). ^{13}C-NMR (75 MHz, $CDCl_3$) δ (ppm):
20.4, 28.9, 34.7, 66.6, 126.4, 126.5, 128.3, 128.6, 129.2, 130.3, 130.9, 135.7, 138.5, 171.6.

benzyl 3-(*p*-tolylthio)propanoate

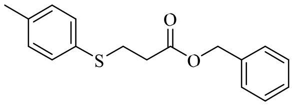

Yellow liquid; Yield: 89% (0.127 g),^{1}H-NMR (300 MHz, $CDCl_3$) δ (ppm): 2.49 (s, 3H, CH_3), 2.57 (t, J = 7.5 Hz, 2H, CH_2), 3.06 (t, J = 7.5 Hz, 2H, CH_2), 5.04 (s, 2H, CH_2), 7.03 (d, J = 9.0 Hz, 1H, Ar), 7.19-7.32 (m, 8H, Ar).

benzyl 3-((4-chlorophenyl)thio)propanoate

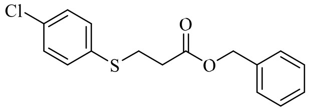

Yellow liquid; Yield: 79% (0.121 g),^{1}H-NMR (300 MHz, $CDCl_3$) δ (ppm): 2.69 (t, J = 7.0 Hz, 2H, CH_2), 3.20 (t, J = 7.0 Hz, 2H, CH_2), 5.16 (s, 2H, CH_2), 7.24-7.57 (m, 9H, Ar). ^{13}C-NMR (75 MHz, $CDCl_3$) δ (ppm): 29.3, 34.3, 66.6, 128.3, 128.4, 128.6, 129.2, 131.6, 132.7, 133.6, 135.6, 171.4.

benzyl 3-((1-methyl-1*H*-imidazol-2-yl)thio)propanoate

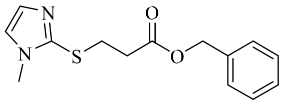

Mild brown solid; Yield: 75% (0.103 g), ^{1}H-NMR (300 MHz, $CDCl_3$) δ (ppm): 2.96 (t, J = 6.0 Hz, 2H, CH_2), 3.60 (s, 3H, CH_3), 4.33 (t, J = 6.0 Hz, 2H, CH_2), 5.13 (s, 2H, CH_2), 6.62 (d, J = 2.5 Hz, 1H, Ar), 6.78 (d, J = 2.5 Hz, 1H, Ar), 7.30-7.37 (m, 5H, Ar). ^{13}C-NMR (75 MHz, $CDCl_3$) δ (ppm): 33.0, 35.0, 43.7, 66.6, 117.4, 117.9, 128.3, 128.4, 128.6, 135.4, 162.1, 171.2.

Anal. Calc. for $C_{14}H_{16}N_2O_2S$: C, 60.85; H, 5.84; N, 10.14; O, 11.58; S, 11.60. Found: C, 60.74; H, 5.80; N, 10.24; S, 11.63.

4-methoxybenzyl 3-(phenylthio)propanoate

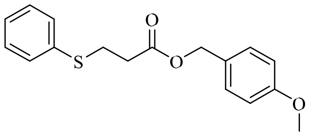

Mild yellow liquid; Yield: 87% (0.131 g), ^{1}H-NMR (300 MHz, $CDCl_3$) δ (ppm): 2.68 (t, J = 7.5 Hz, 2H, CH_2), 3.21 (t, J = 7.5 Hz, 2H, CH_2), 3.84 (s, 3H, CH_3), 5.09 (s, 2H, CH_2), 6.91-6.94 (m, 2H, Ar), 7.21-7.94
(m, 7H, Ar). ^{13}C-NMR (75 MHz, $CDCl_3$) δ (ppm): 29.0, 34.5, 55.3, 66.4, 113.9, 126.6, 127.8, 129.0, 130.2, 130.2, 135.1, 159.7, 171.7.

Anal. Calc. for $C_{17}H_{18}O_3S$: C, 67.52; H, 6.00; O, 15.87; S, 10.60. Found: C, 67.43; H, 6.12; S, 10.65.

4-methoxybenzyl 3-(benzylthio)propanoate

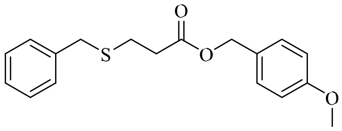

Yellow solid; Yield: 94% (0.148 g), ^{1}H-NMR (300 MHz, $CDCl_3$) δ (ppm): 2.62 (t, J = 6.5 Hz, 2H, CH_2), 2.74 (t, J = 6.5 Hz, 2H, CH_2), 3.76 (s, 2H, CH_2), 3.85 (s, 3H, CH_3), 5.10 (s, 2H, CH_2), 6.91-6.96 (m, 2H, Ar), 7.26-7.38 (m, 7H, Ar). ^{13}C-NMR (75 MHz, $CDCl_3$) δ (ppm): 26.2, 34.6, 36.3, 55.3, 66.3, 113.8, 127.1, 128.5, 128.8, 130.1, 132.3, 138.0, 159.7, 171.8.

Anal. Calc. for $C_{18}H_{20}O_3S$: C, 68.33; H, 6.37; O, 15.17; S, 10.13. Found: C, 68.35; H, 6.45; S, 10.09.

4-methoxybenzyl 3-((4-chlorophenyl)thio)propanoate

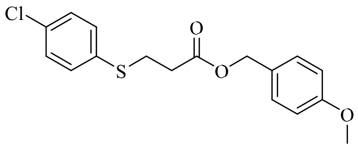

Yellow oily liquid; Yield: 82% (0.138 g), ^{1}H-NMR (300 MHz, $CDCl_3$) δ (ppm): 2.66 (t, J = 7.5 Hz, 2H, CH_2), 3.18 (t, J = 7.5 Hz, 2H, CH_2), 4.50 (s, 3H, CH_3), 5.09 (s, 2H, CH_2), 6.88-6.96 (m, 4H, Ar), 7.23-7.33 (m, 4H, Ar). ^{13}C-NMR (75 MHz, $CDCl_3$) δ (ppm): 29.3, 34.3, 55.3, 66.5, 113.7, 127.7, 129.1, 129.4, 130.2, 130.4, 131.5, 159.1, 171.5.

Anal. Calc. for $C_{17}H_{17}ClO_3S$: C, 60.62; H, 5.09; Cl, 10.52; O, 14.25; S, 9.52. Found: C, 60.71; H, 5.15; S, 9.50.

4-nitrobenzyl 3-((4-bromophenyl)thio)propanoate

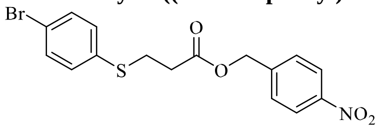

Brown solid; Yield: 70% (0.138 g), ^{1}H-NMR (300 MHz, $CDCl_3$) δ (ppm): 2.62 (t, J = 7.0 Hz, 2H, CH_2), 3.08 (t, J = 7.0 Hz, 2H, CH_2), 5.11 (s, 2H, CH_2), 7.11 (d, J = 8.5 Hz, 2H, CH, Ar), 7.29 (d, J = 8.5 Hz, 2H, CH, Ar), 7.40 (d, J = 8.5 Hz, 2H, CH, Ar), 8.09 (d, J = 8.5 Hz, 2H, CH, Ar). ^{13}C-NMR (75 MHz, $CDCl_3$) δ (ppm): 29.0, 34.1, 65,1. 120.6, 123.8, 128.4, 131.6, 132.1, 134.3, 142.9, 147.6, 171.1.

Anal. Calc. for $C_{16}H_{14}BrNO_4S$: C, 48.50; H, 3.56; Br, 20.16; N, 3.53; O, 16.15; S, 8.09. Found: C, 48.48; H, 3.63; N, 3.59; S, 8.00.

4-nitrobenzyl 3-(octylthio)propanoate

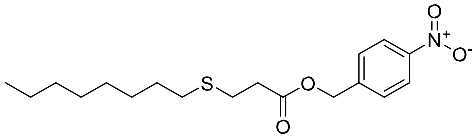

Dark brown liquid; Yield: 76% (0.134 g), ^{1}H-NMR (300 MHz, $CDCl_3$) δ (ppm): 0.80 (t, J = 6.0 Hz, 3H, CH_3), 1.18-1.31 (m, 10H, CH_2), 1.50 (p, J = 7.5 Hz, 2H, CH_2), 2.45 (t, J = 7.5 Hz, 2H, CH_2), 2.63 (t, J = 7.0 Hz, 2H, CH_2), 2.74 (t, J = 7.0 Hz, 2H, CH_2), 5.17 (s, 2H, CH_2), 7.46 (d, J = 8.5 Hz, 2H, CH, Ar), 8.16 (d, J = 8.5 Hz, 2H, CH, Ar). ^{13}C-NMR (75 MHz, $CDCl_3$) δ (ppm): 14.1, 22.6, 26.9, 28.8, 29.0, 29.2, 29.5, 31.8, 32.2, 34.7, 64.9, 123.8, 128.4, 130.7, 143.0, 171.6.

Anal. Calc. for $C_{18}H_{27}NO_4S$: C, 61.16; H, 7.70; N, 3.96; O, 18.10; S, 9.07. Found: C, 61.19; H, 7.79; N, 3.99; S, 8.99.

1-phenylethyl 3-((4-chlorophenyl)thio)propanoate

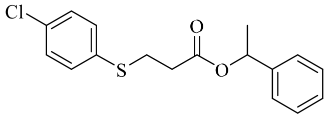

Mild yellow liquid; Yield: 68% (0.109 g), ^{1}H-NMR (300 MHz, $CDCl_3$) δ (ppm): 1.59 (d, J = 6.5 Hz, 3H, CH_3), 2.68 (td, J_1 = 7.5 Hz, J_2 = 3.0 Hz, 2H, CH_2), 3.18 (t, J = 7.5 Hz, 2H, CH_2), 5.95 (q, 1H, CH), 7.277.40 (m, 9H, Ar). ^{13}C-NMR (75 MHz, $CDCl_3$) δ (ppm): 22.2, 29.3, 34.5, 72.9, 126.1, 128.0, 128.5, 129.2, 131.5, 132.6, 133.8, 141.3, 170.8.

Anal. Calc. for $C_{17}H_{17}ClO_2S$: C, 63.64; H, 5.34; Cl, 11.05; O, 9.97; S, 9.99. Found: C, 63.71; H, 5.40; S, 10.02.

octyl 3-(phenylthio)propanoate

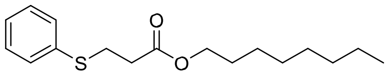

Yellow liquid; Yield: 79% (0.116 g), ^{1}H-NMR (300 MHz, $CDCl_3$) δ (ppm): 0.91 (t, J = 6.0 Hz, 3H, CH_3), 1.21-1.36 (m, 11H, CH_2), 1.47-1.66 (m, 2H, CH_2), 2.61 (t, J = 7.5 Hz, 2H, CH_2), 3.17 (t, J = 7.5 Hz, 2H, CH_2), 4.96 (six, J = 6.0 Hz, 2H, CH_2), 7.24-7.35 (m, 5H, Ar). ^{13}C-NMR (75 MHz, $CDCl_3$) δ (ppm): 14.1, 22.6, 25.9, 28.5, 29.1, 29.1, 31.7, 34.4, 38.1, 64.9, 126.5, 129.0, 130.1, 131.2, 171.9.

octan-2-yl 3-((4-chlorophenyl)thio)propanoate

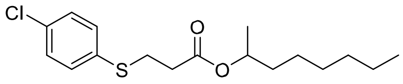

Yellow liquid; Yield: 70% (0.115 g), ^{1}H-NMR (300 MHz, $CDCl_3$) δ (ppm): 1.21-1.66 (m, 16H, CH_2-CH_3), 2.61 (t, J = 7.5 Hz, 2H, CH_2), 3.17 (t, J = 7.5 Hz, 2H, CH_2), 4.94 (six, J = 6.0 Hz, 1H, CH), 7.24-7.35 (m, 4H, Ar). ^{13}C-NMR (75 MHz, $CDCl_3$) δ (ppm): 14.0, 19.9, 22.5, 25.3, 29.1, 29.4, 31.7, 34.5, 35.9, 71.7, 129.1, 131.4, 132.6, 133.9, 171.2.

Anal. Calc. for $C_{17}H_{25}ClO_2S$: C, 62.08; H, 7.66; Cl, 10.78; O, 9.73; S, 9.75. Found: C, 62.09; H, 7.60; S, 9.73.

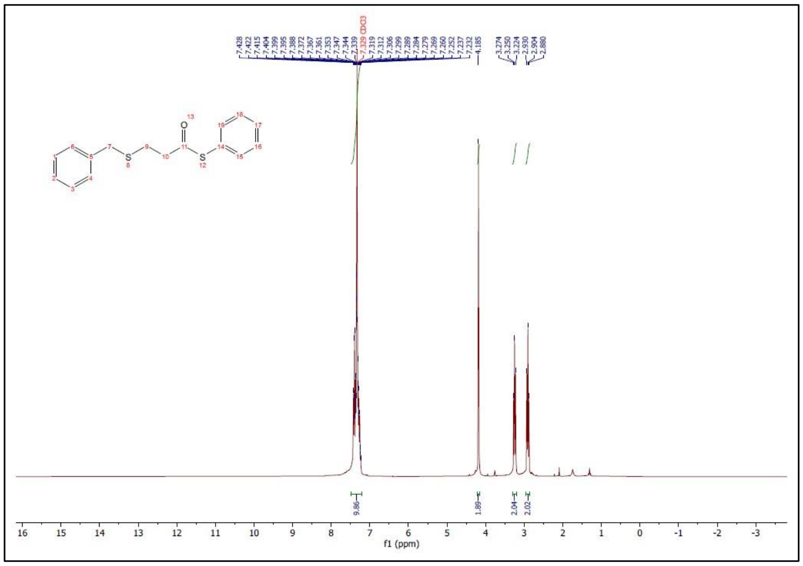

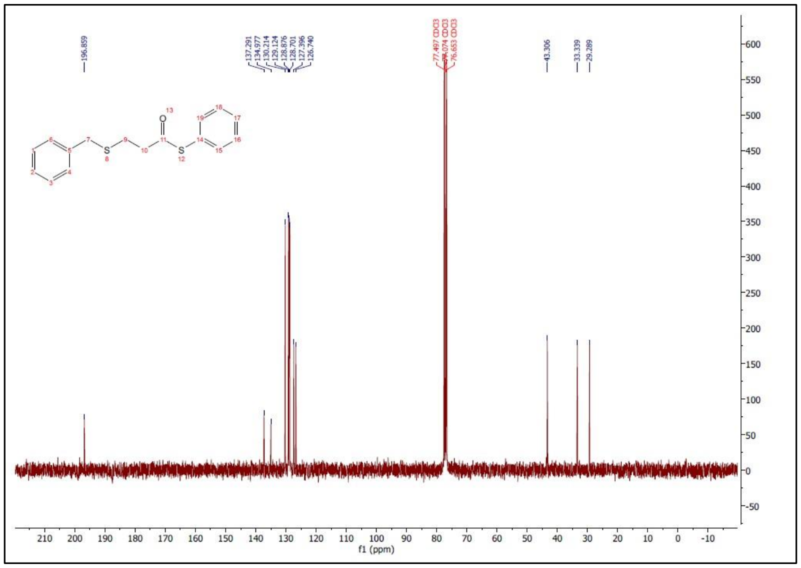

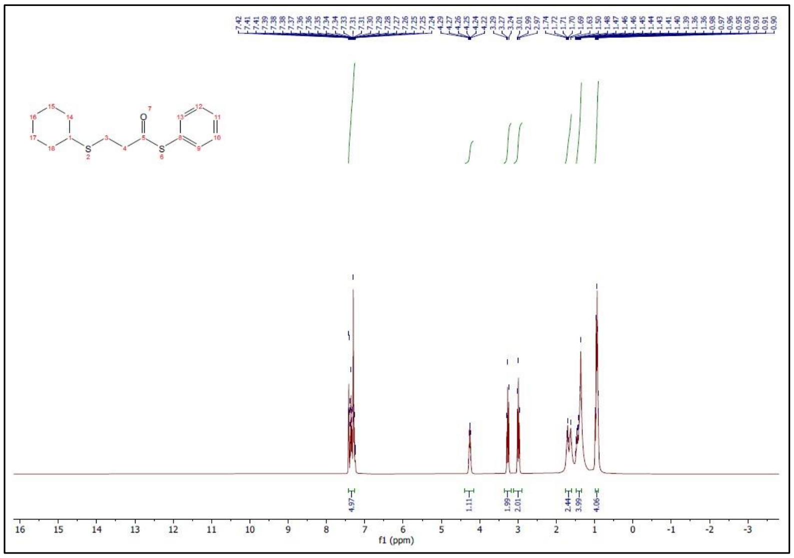
f1 (ppm)

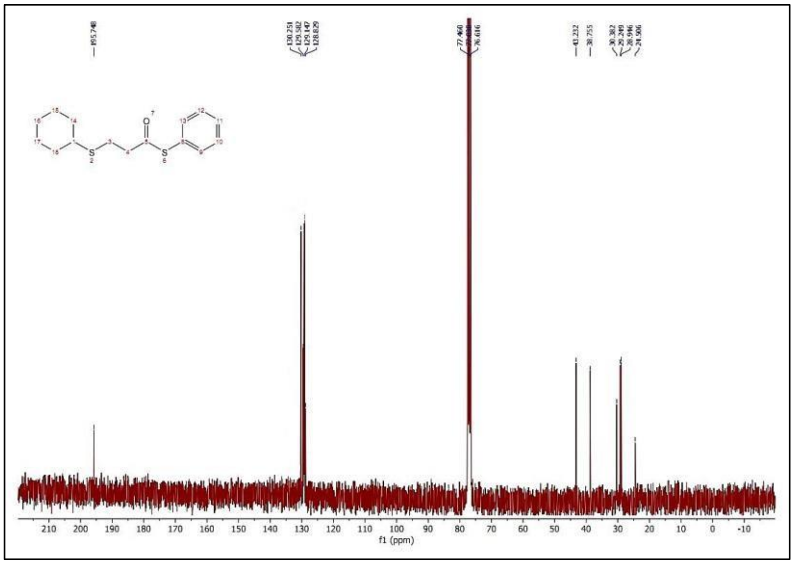
f1 (ppm)

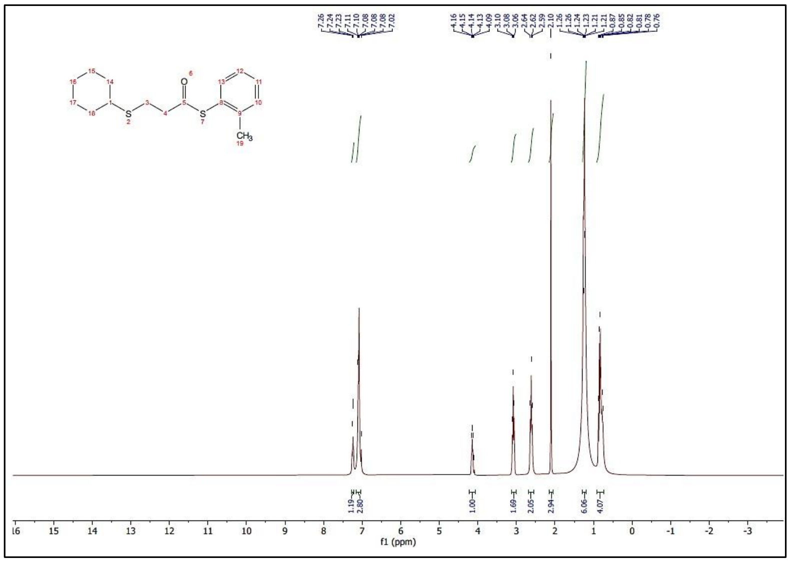

f1 (ppm)
CH3

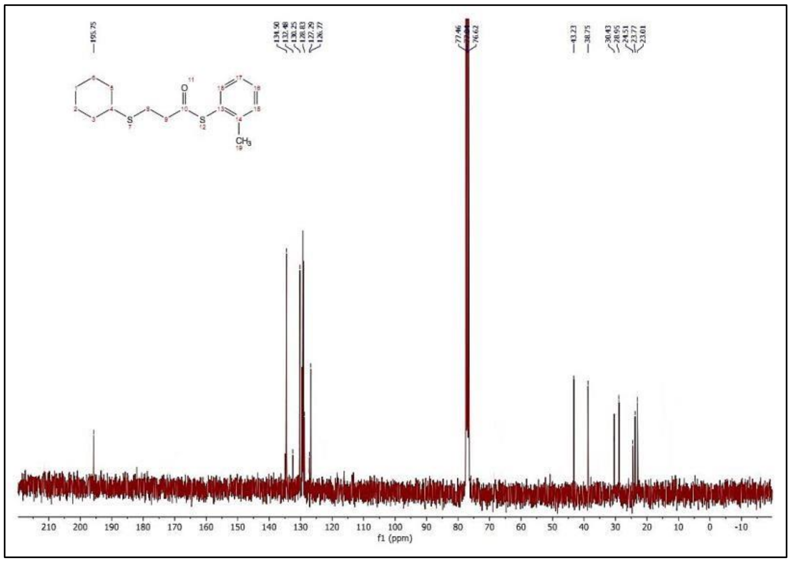

f1 (ppm)
CH3

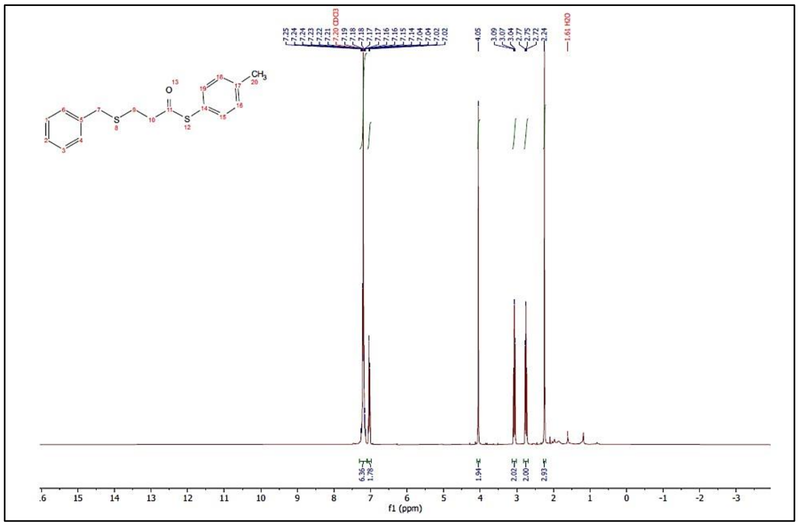

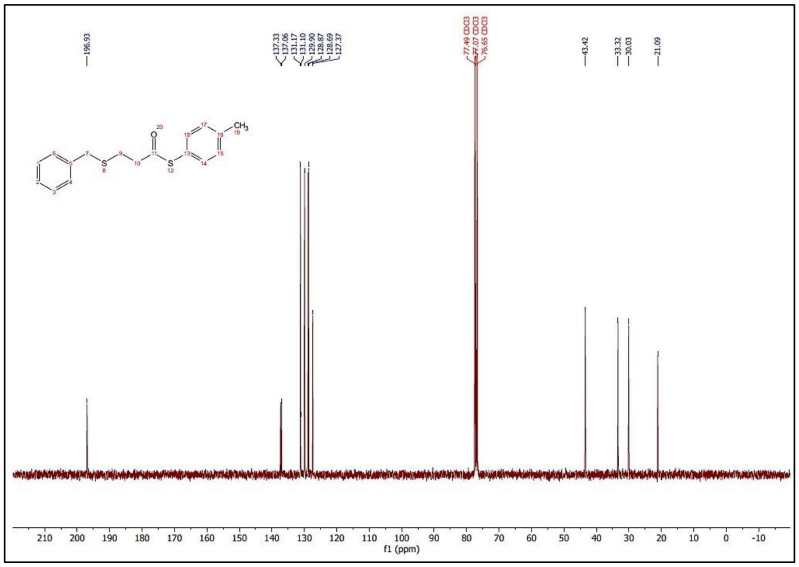

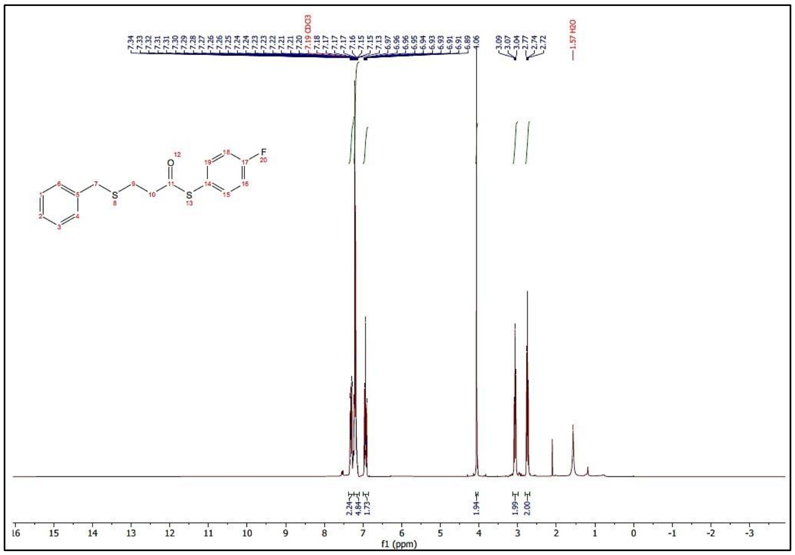

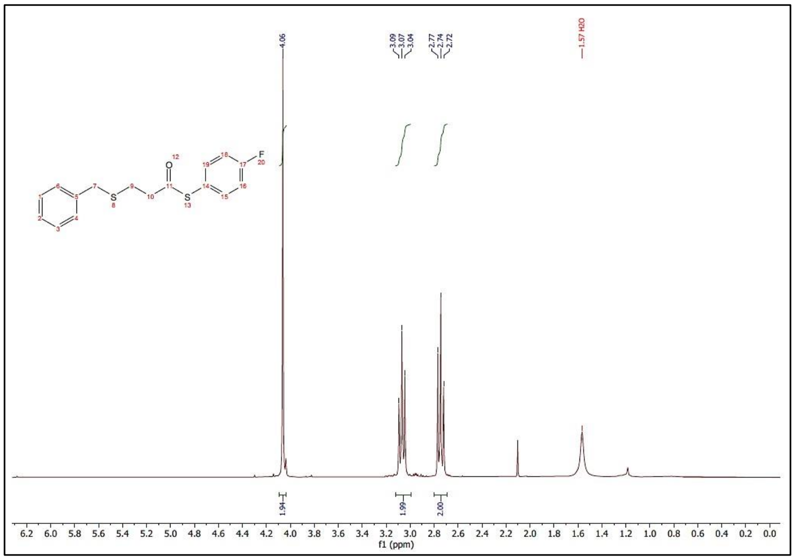

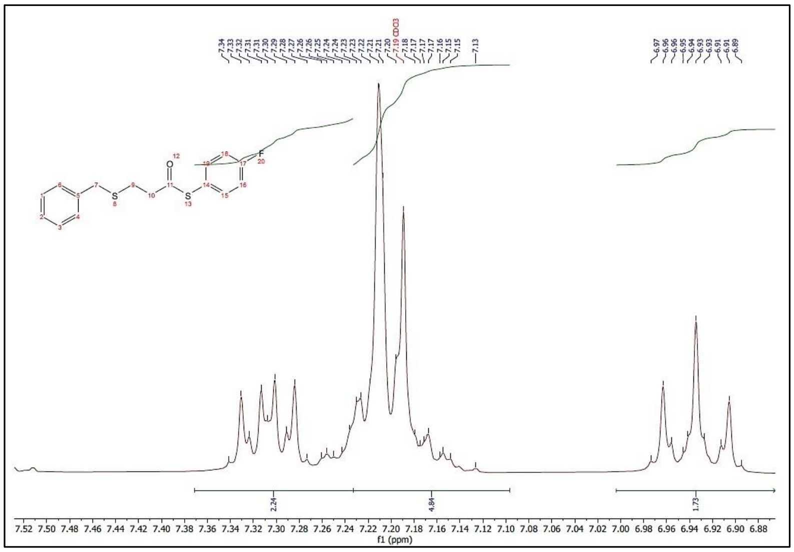

7.34
7.33
7.32
7.31
7.31
7.30
7.29
7.28
7.27
7.26
7.26
7.25
7.24
7.24
7.23
7.23
7.22
7.21
7.21
7.20
7.19 CDCl3
7.18
7.17
7.17
7.17
7.16
7.15
7.13
6.97
6.96
6.96
6.95
6.94
6.93
6.93
6.91
6.91
6.89
2.24
4.84
1.73
f1 (ppm)

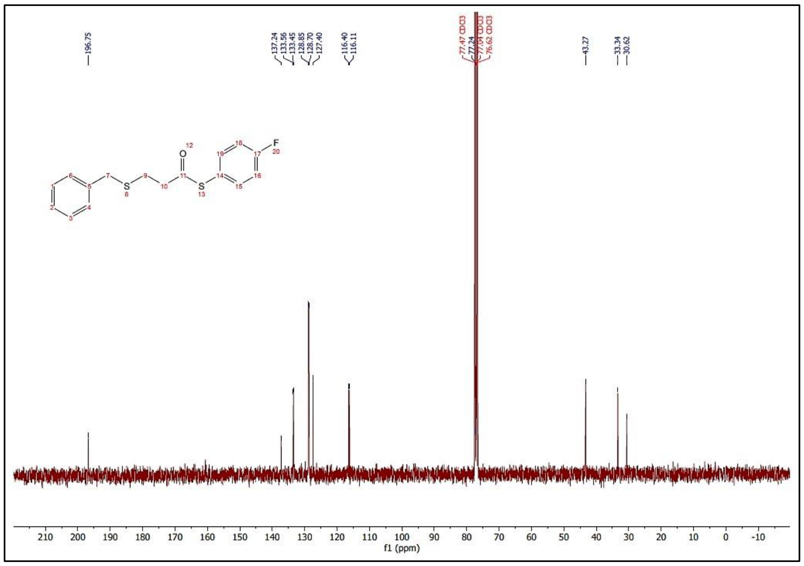

196.75
137.24
133.56
133.45
128.85
128.70
127.40
116.40
116.11
77.47 CDCl3
77.24
77.04 CDCl3
76.62 CDCl3
43.27
33.34
30.62
f1 (ppm)

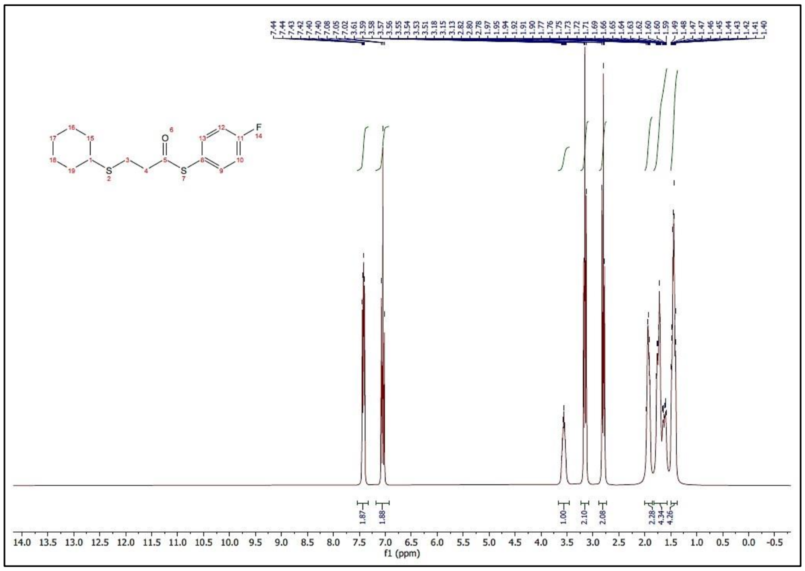
F
1.87
1.88
1.00
2.10
2.08
2.28
4.34
4.26
14.0 13.5 13.0 12.5 12.0 11.5 11.0 10.5 10.0 9.5 9.0 8.5 8.0 7.5 7.0 6.5 6.0 5.5 5.0 4.5 4.0 3.5 3.0 2.5 2.0 1.5 1.0 0.5 0.0 -0.5
f1 (ppm)

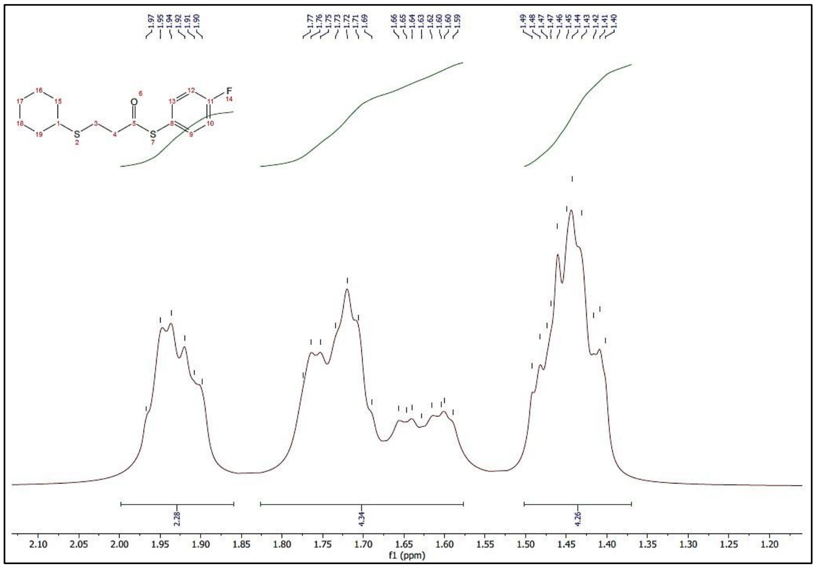
1.97
1.95
1.94
1.92
1.91
1.90
1.77
1.76
1.75
1.73
1.72
1.71
1.69
1.66
1.65
1.64
1.63
1.62
1.60
1.60
1.59
1.49
1.48
1.47
1.46
1.45
1.44
1.43
1.42
1.41
1.40
F
2.28
4.34
4.26
2.10 2.05 2.00 1.95 1.90 1.85 1.80 1.75 1.70 1.65 1.60 1.55 1.50 1.45 1.40 1.35 1.30 1.25 1.20
f1 (ppm)

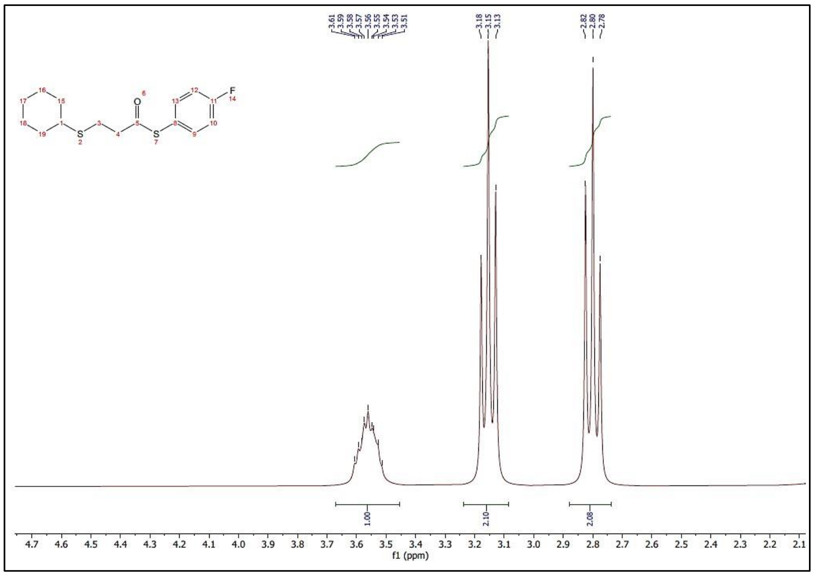

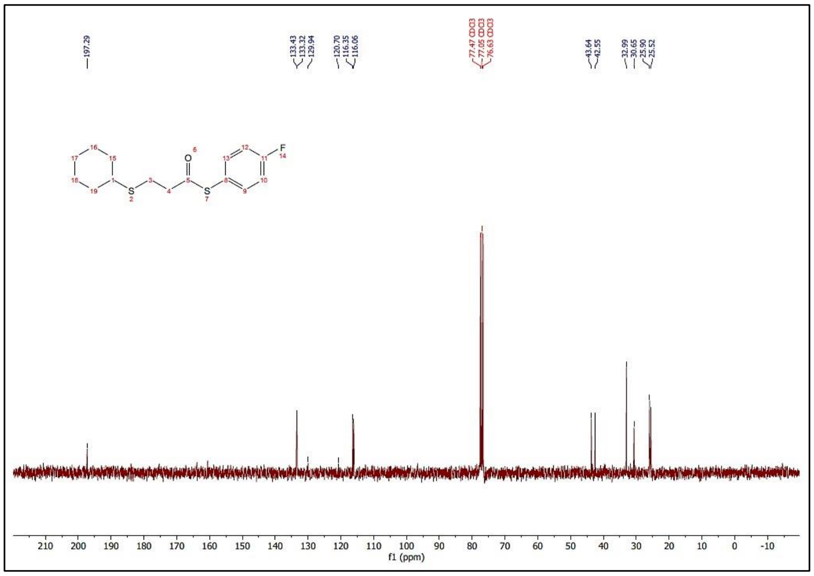

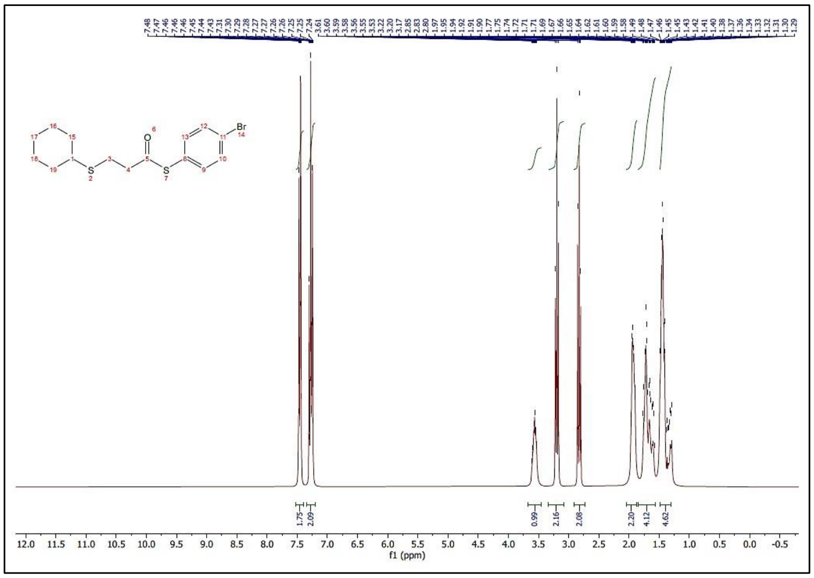

Br
f1 (ppm)

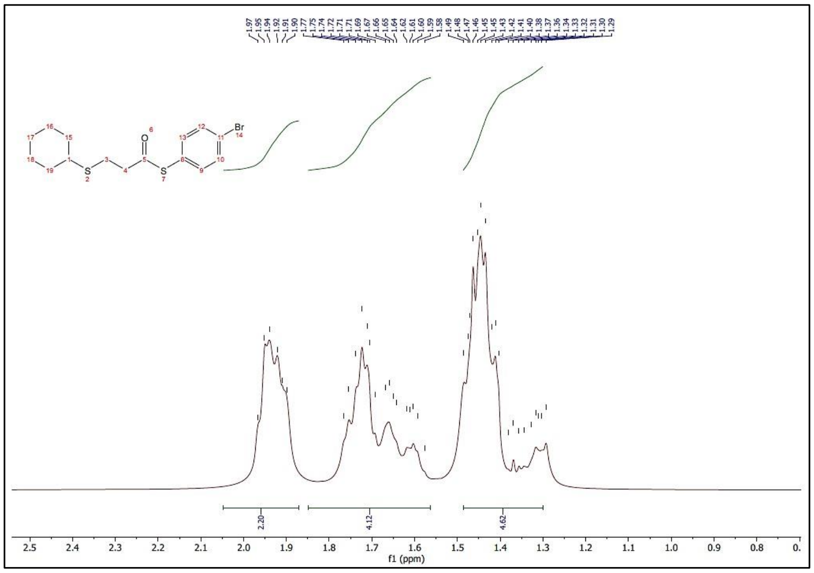

Br
f1 (ppm)

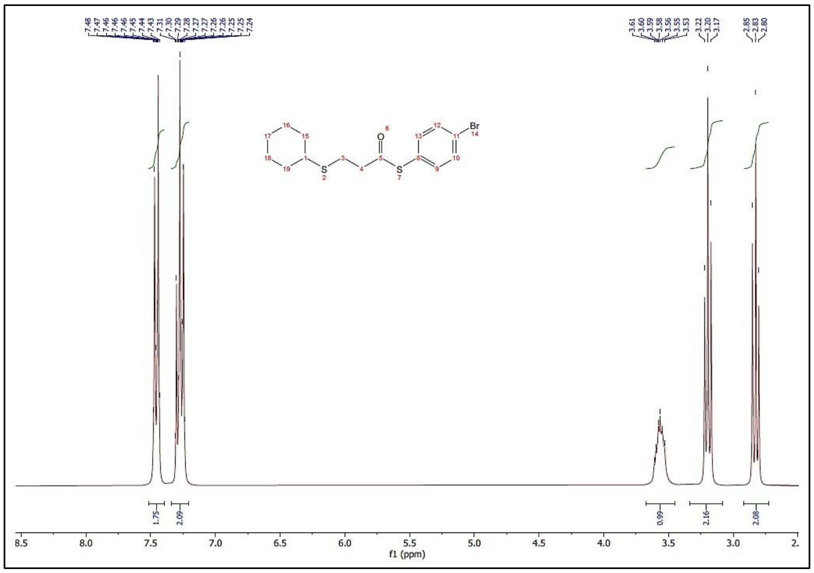

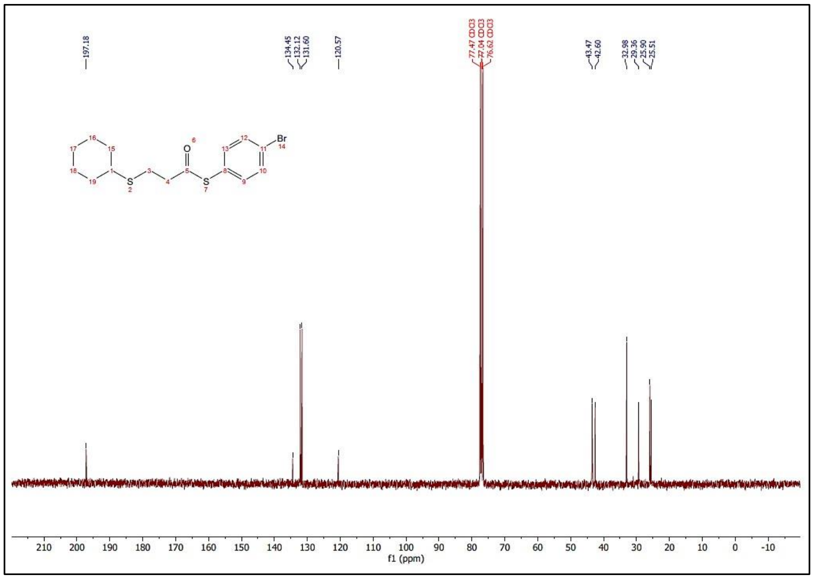

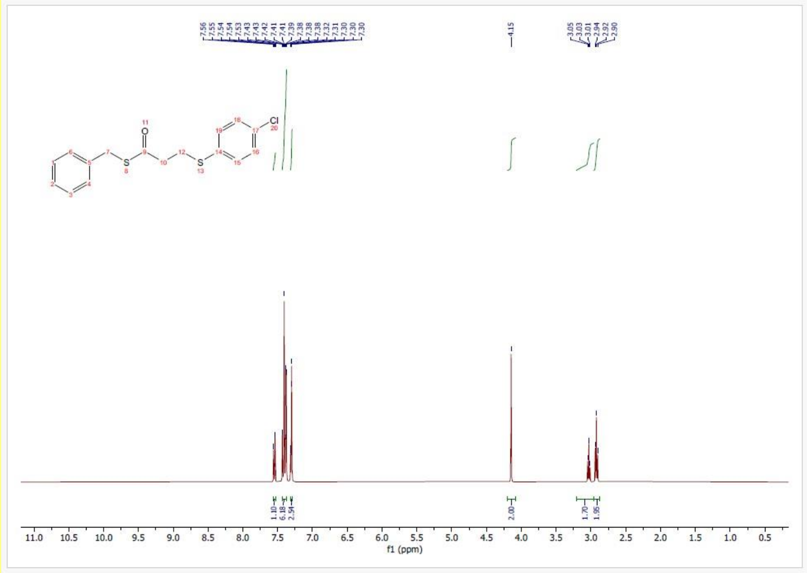

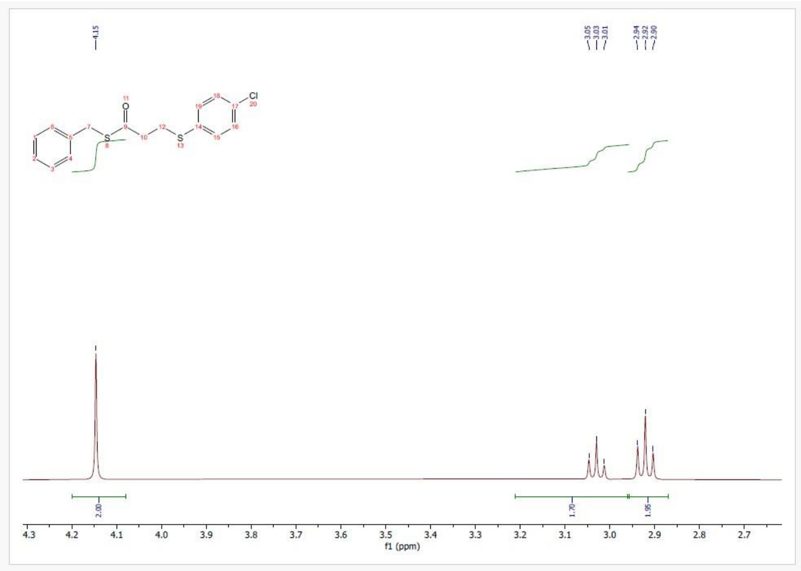

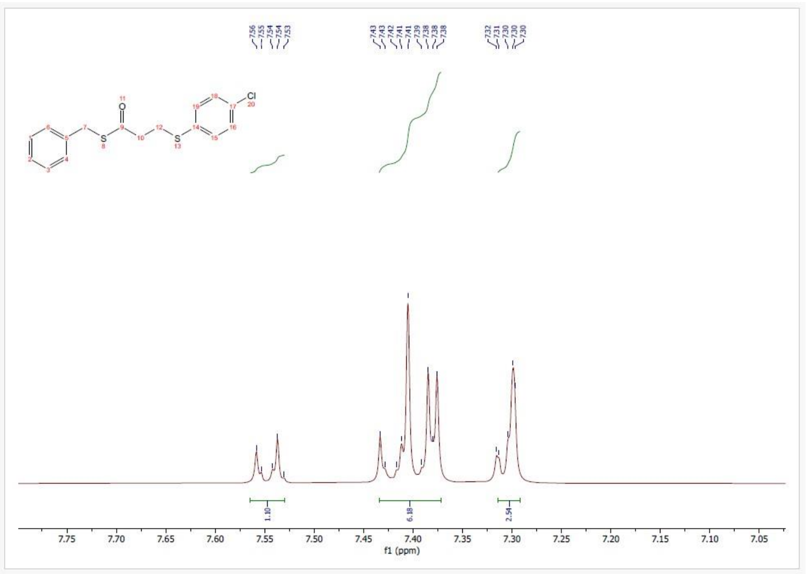

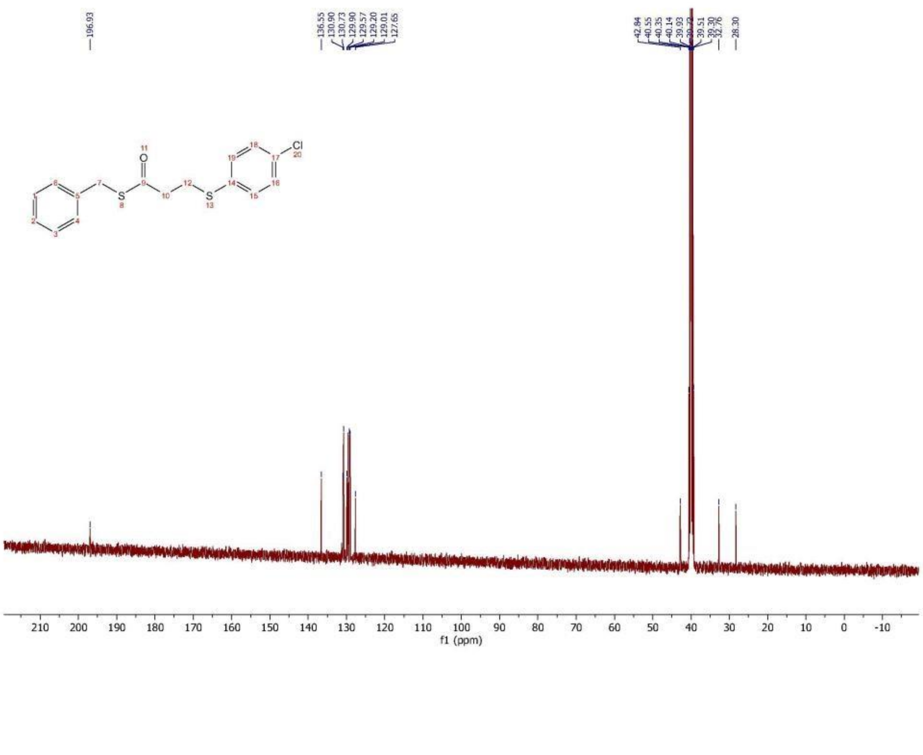

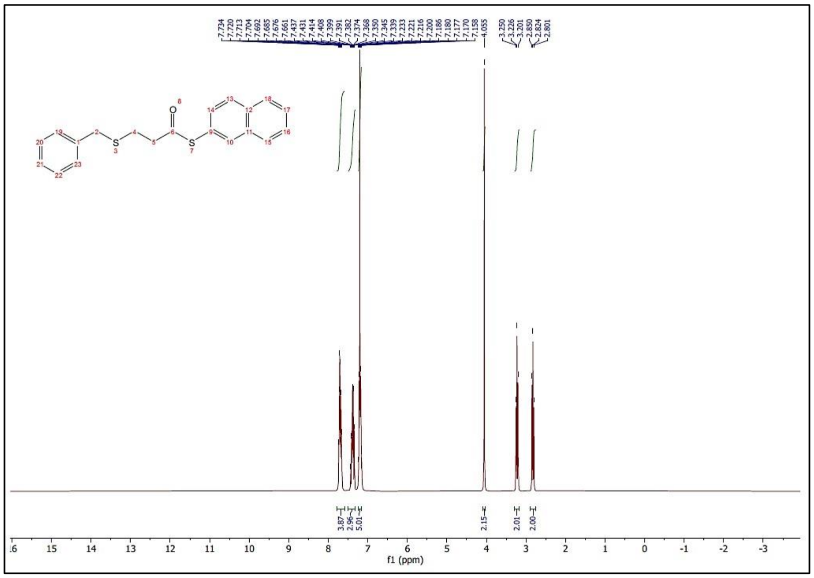

7.734
7.720
7.713
7.704
7.692
7.685
7.676
7.661
7.437
7.431
7.414
7.408
7.399
7.391
7.382
7.374
7.368
7.350
7.345
7.339
7.233
7.221
7.216
7.200
7.186
7.180
7.177
7.170
7.158
4.055
3.250
3.226
3.201
2.850
2.824
2.801
3.87
2.96
5.01
2.15
2.01
2.00
f1 (ppm)

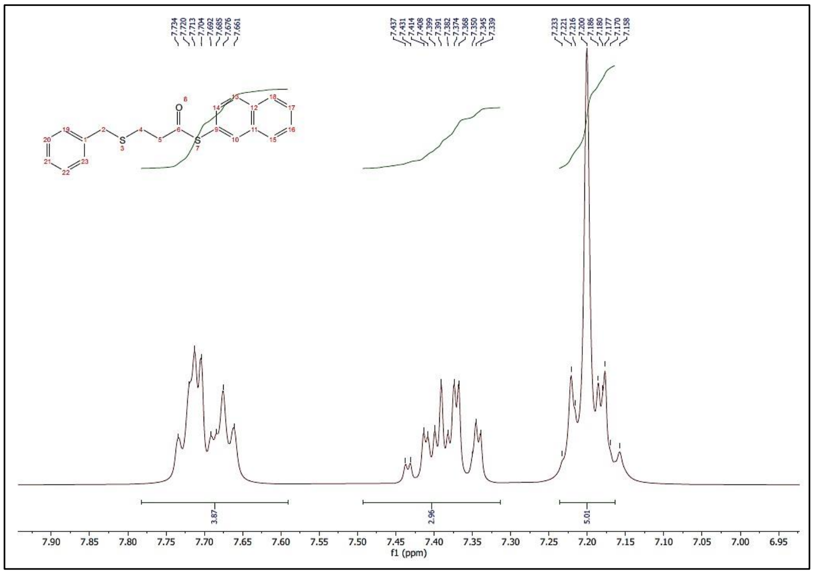

7.734
7.720
7.713
7.704
7.692
7.685
7.676
7.661
7.437
7.431
7.414
7.408
7.399
7.391
7.382
7.374
7.368
7.350
7.345
7.339
7.233
7.221
7.216
7.200
7.186
7.180
7.177
7.170
7.158
3.87
2.96
5.01
f1 (ppm)

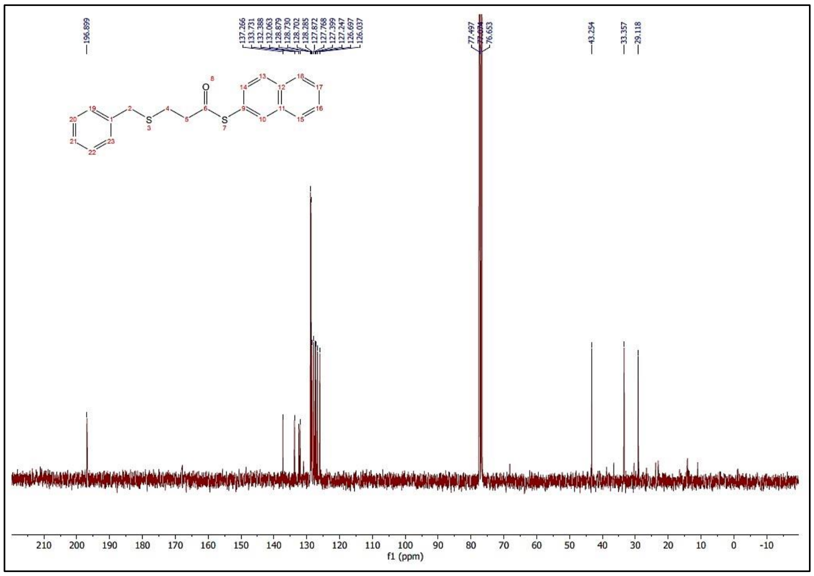
196.899
137.266
133.731
133.388
132.063
128.879
128.750
128.702
128.285
127.872
127.768
127.399
127.247
126.697
126.037
77.497
76.653
43.254
33.357
29.118
f1 (ppm)

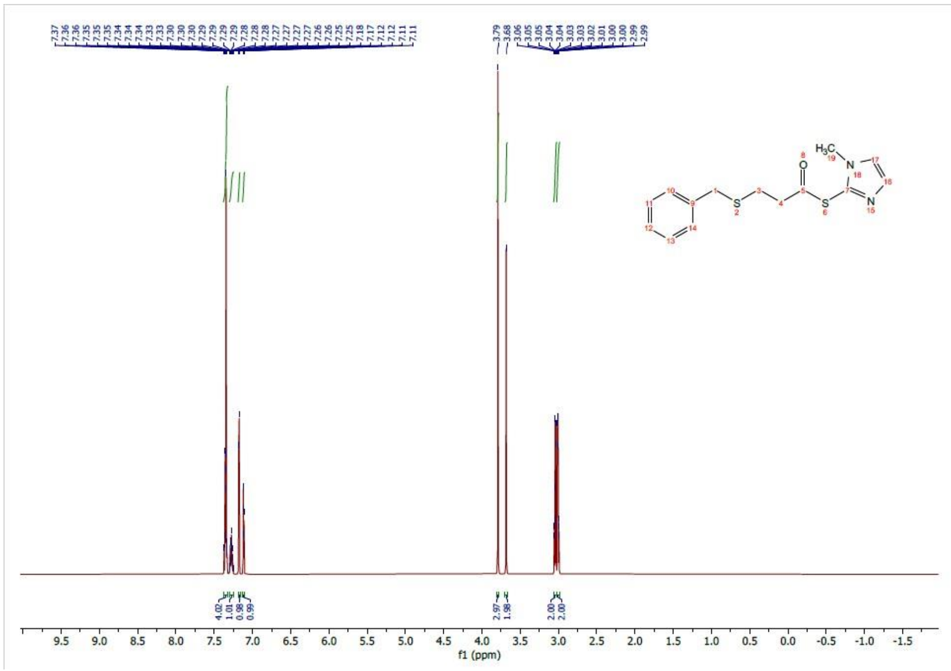
3.79
3.68
4.02
1.01
0.98
0.99
2.97
1.98
2.00
2.00
H3C
N
f1 (ppm)

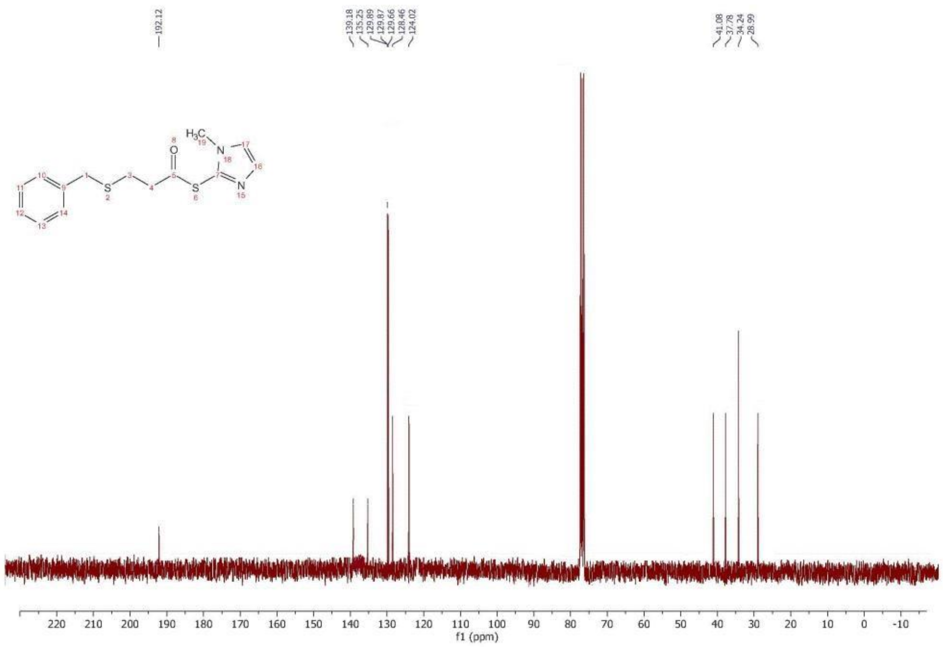

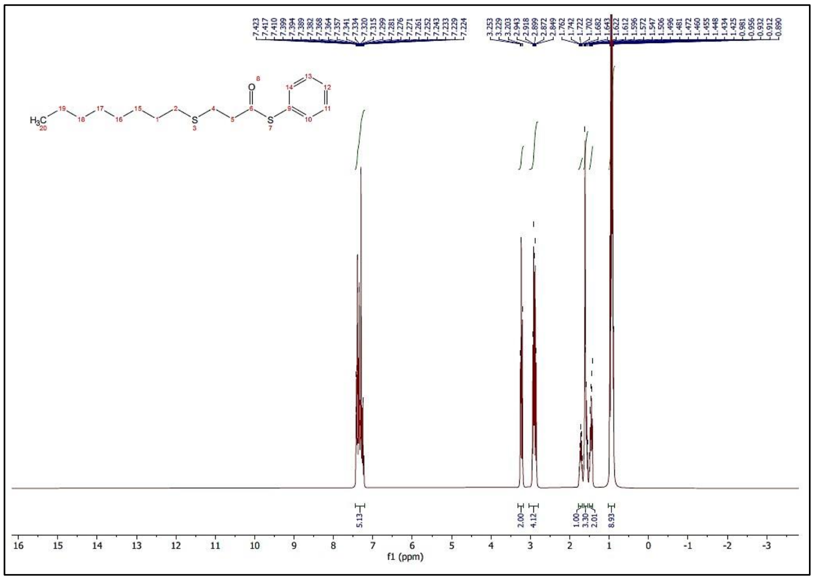

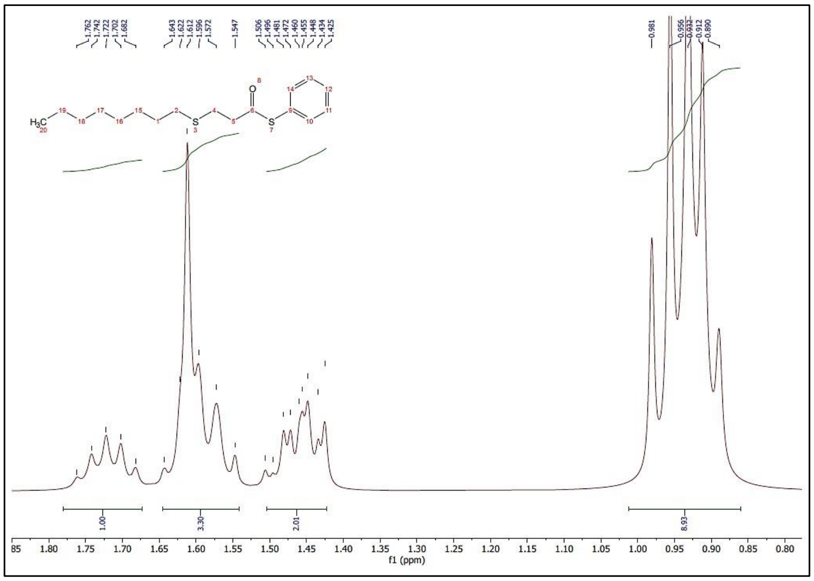

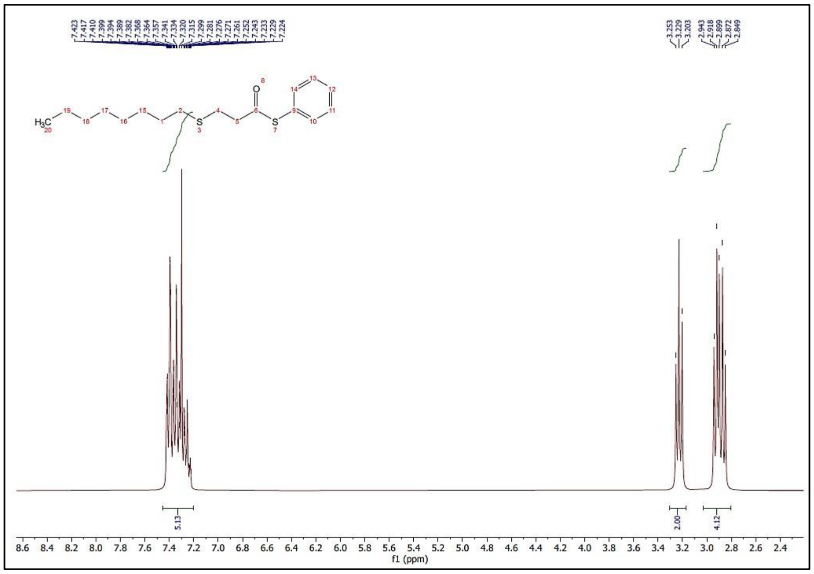

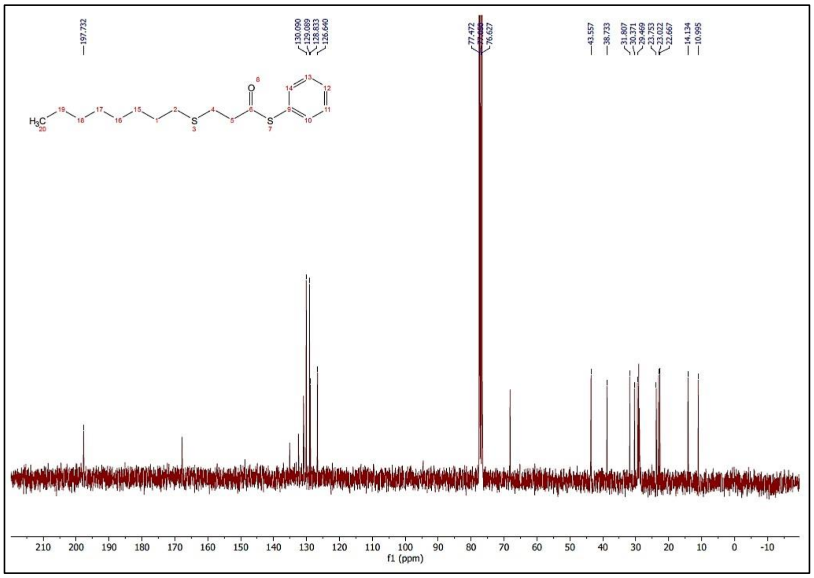
f1 (ppm)

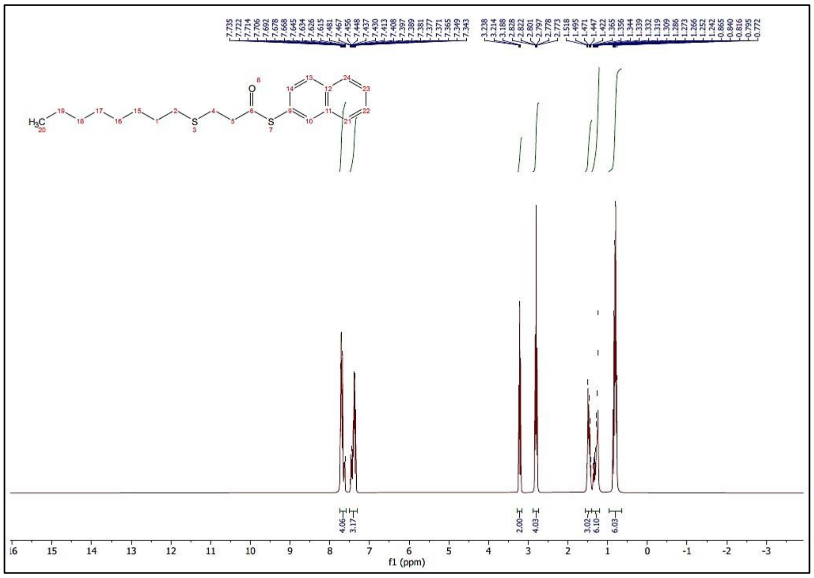
f1 (ppm)

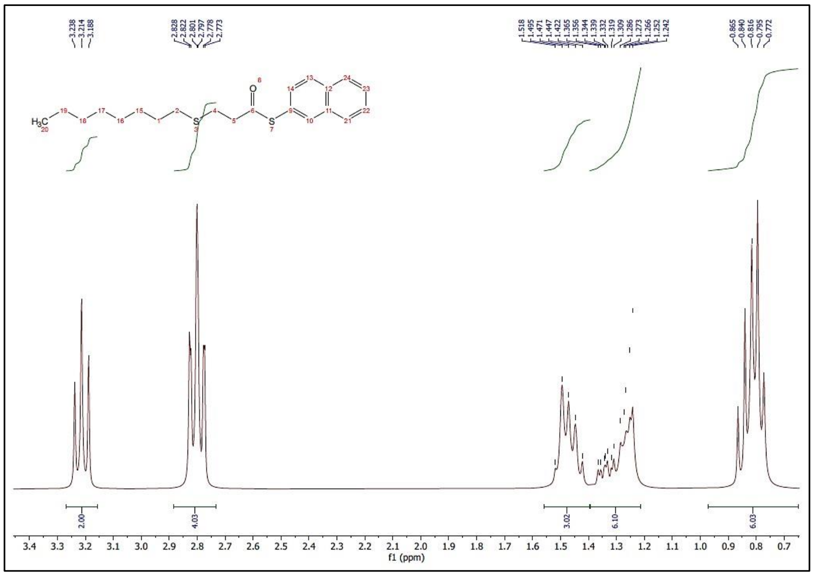
f1 (ppm)

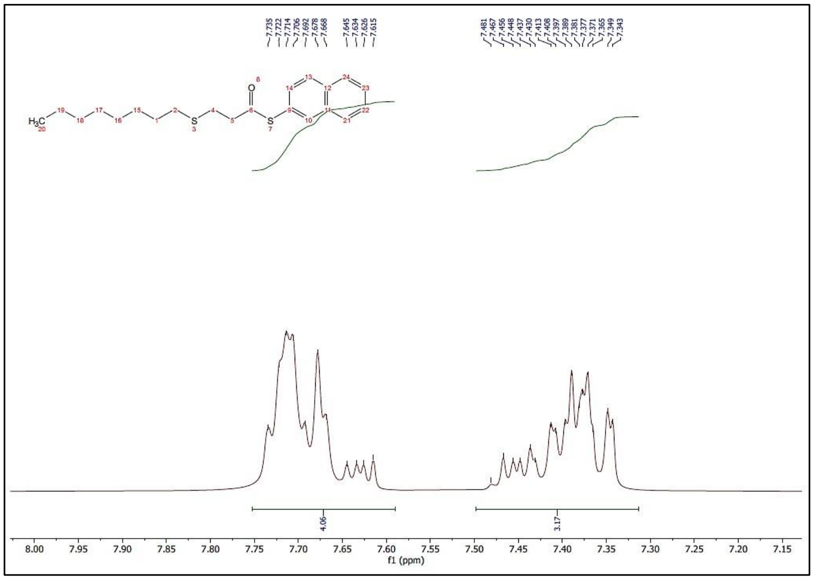
f1 (ppm)

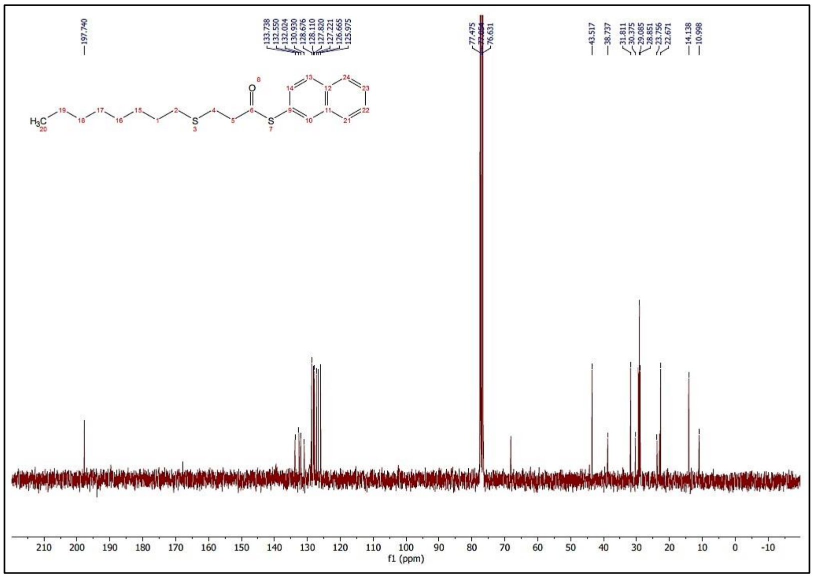
197.740
133.738
132.550
132.024
130.930
128.676
128.110
127.820
127.221
126.665
125.975
77.475
76.631
43.517
38.737
31.811
30.375
29.085
28.851
23.756
22.671
14.138
10.998
f1 (ppm)

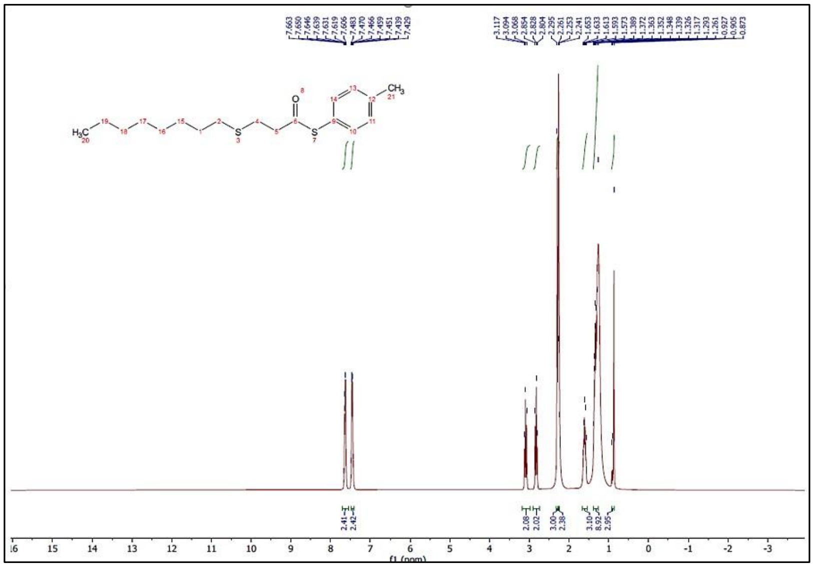
7.663
7.650
7.646
7.639
7.631
7.619
7.606
7.483
7.470
7.466
7.459
7.451
7.439
7.429
3.117
3.094
3.068
2.854
2.828
2.804
2.295
2.261
2.253
2.241
1.653
1.633
1.613
1.593
1.573
1.389
1.372
1.363
1.352
1.348
1.339
1.326
1.317
1.293
1.261
0.927
0.905
0.873
2.41
2.42
2.08
2.02
3.00
2.38
3.10
8.92
2.95
f1 (ppm)

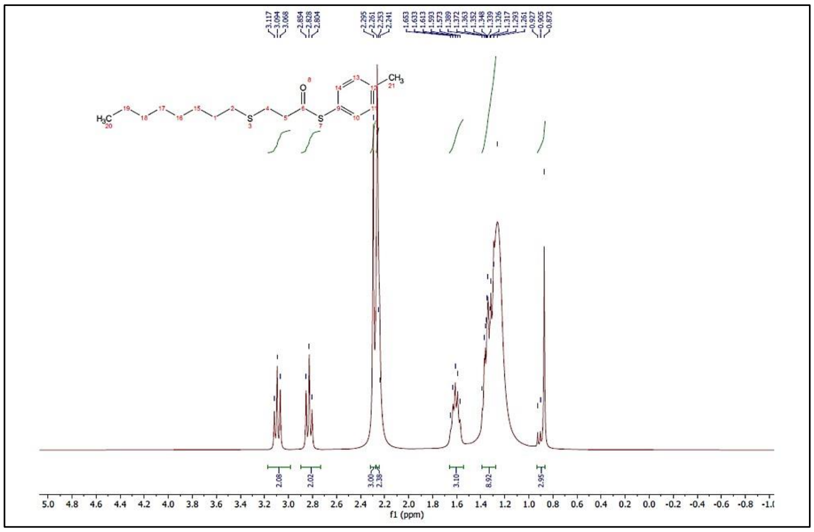

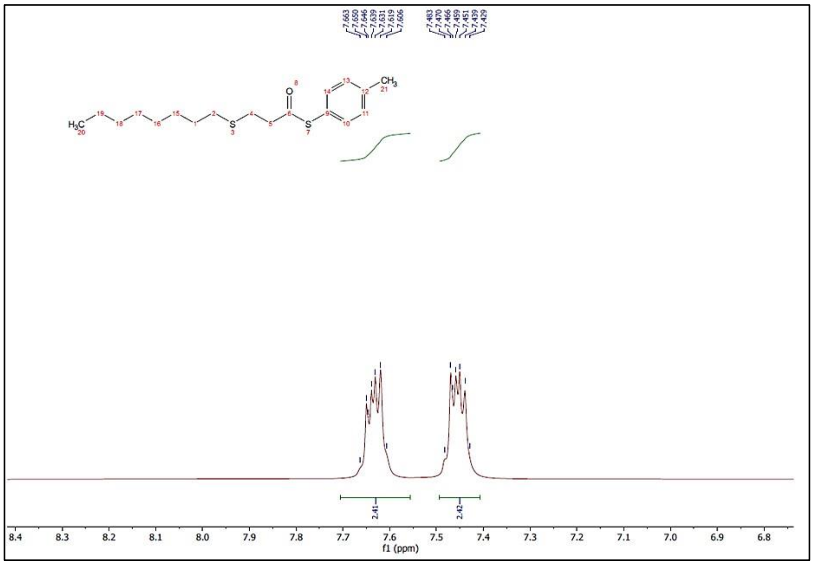

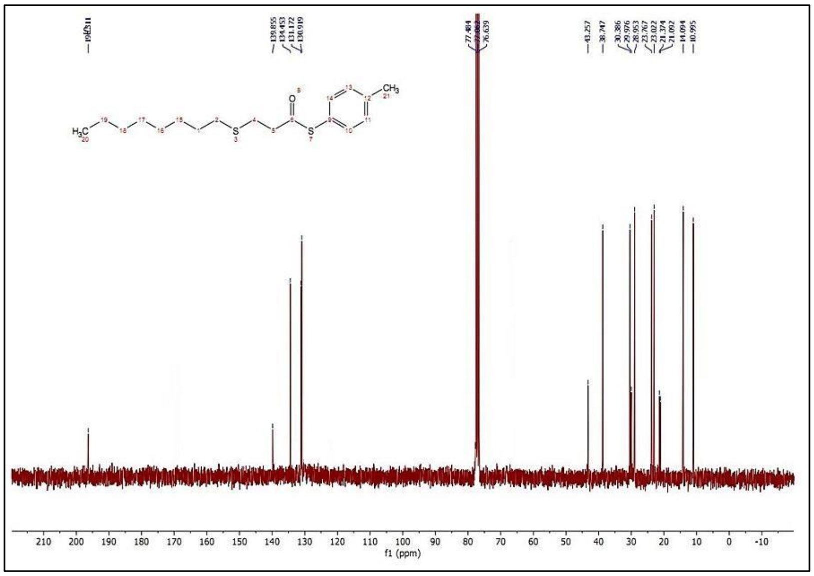

f1 (ppm)

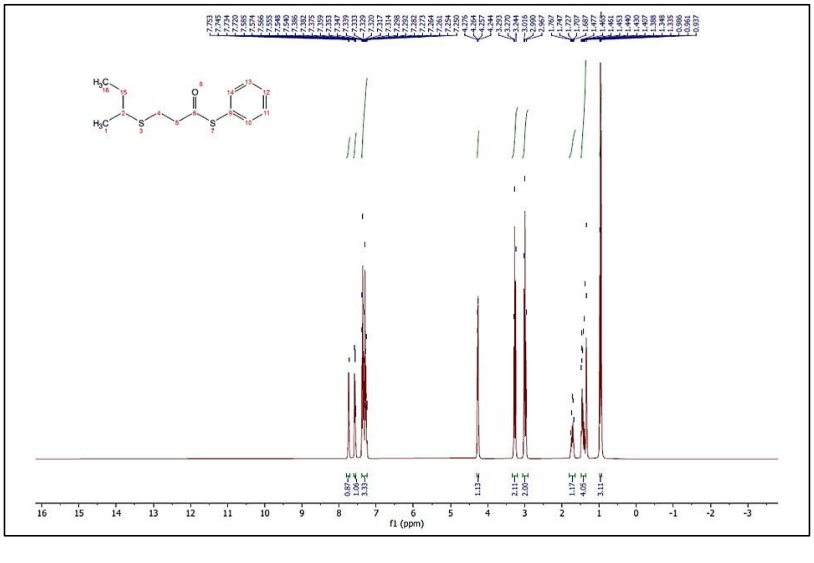

f1 (ppm)

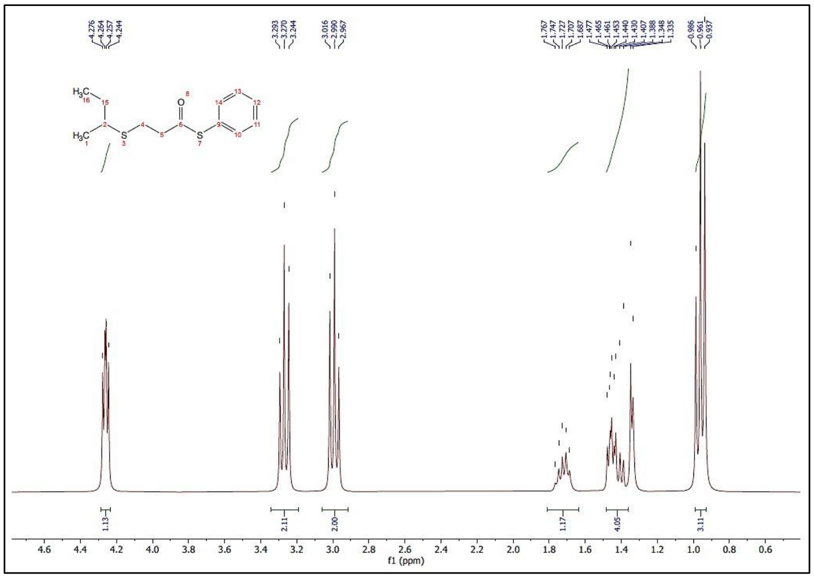

f1 (ppm)

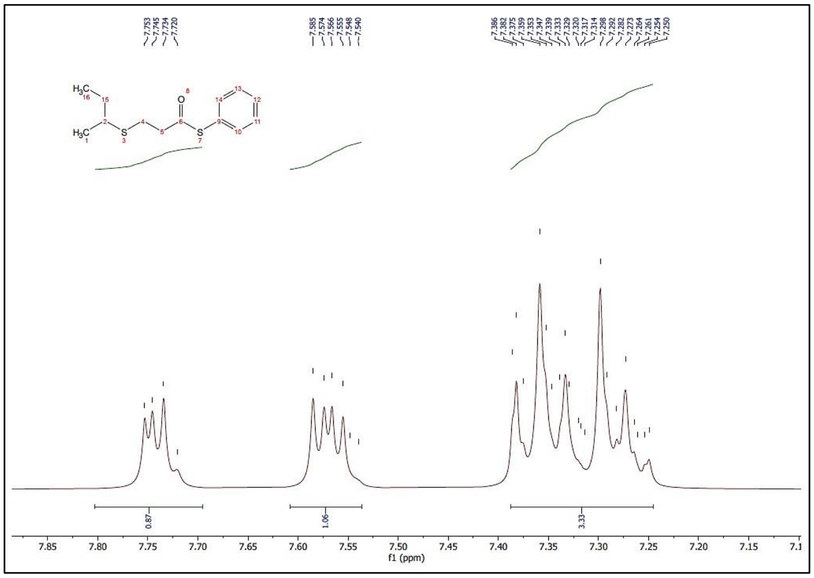

f1 (ppm)

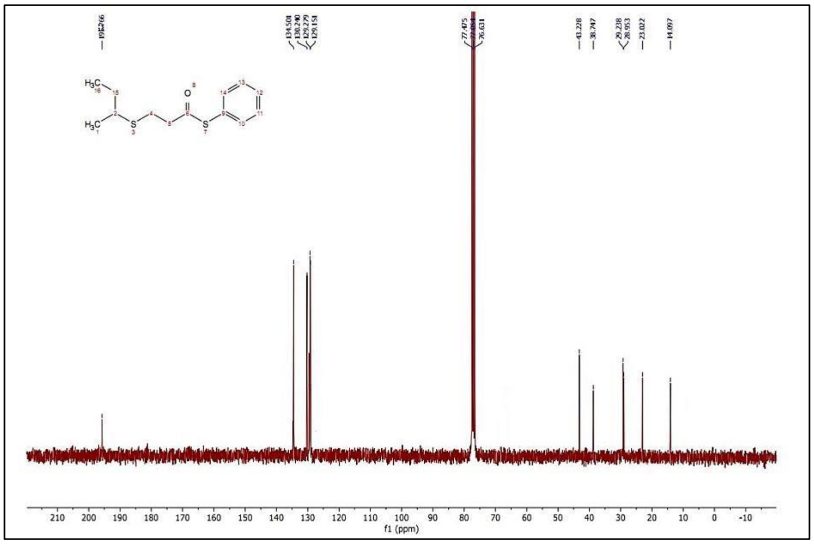

134.501
130.240
129.279
129.151
77.475
76.631
43.228
38.747
29.238
28.953
23.022
14.097
f1 (ppm)

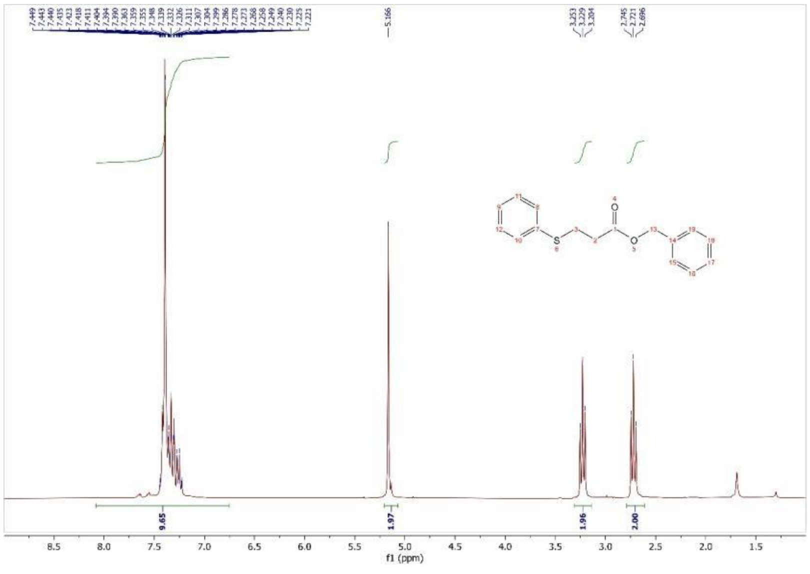

7.449
7.443
7.440
7.435
7.423
7.418
7.411
7.404
7.394
7.390
7.363
7.359
7.355
7.348
7.339
7.332
7.326
7.311
7.307
7.304
7.299
7.286
7.278
7.273
7.268
7.258
7.249
7.240
7.230
7.225
7.221
5.166
3.253
3.229
3.204
2.745
2.721
2.696
9.65
1.97
1.96
2.00
f1 (ppm)

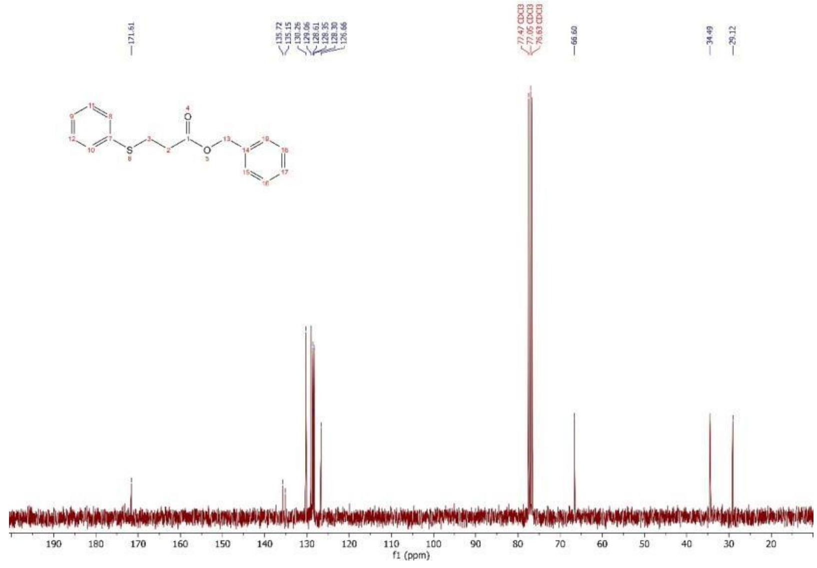

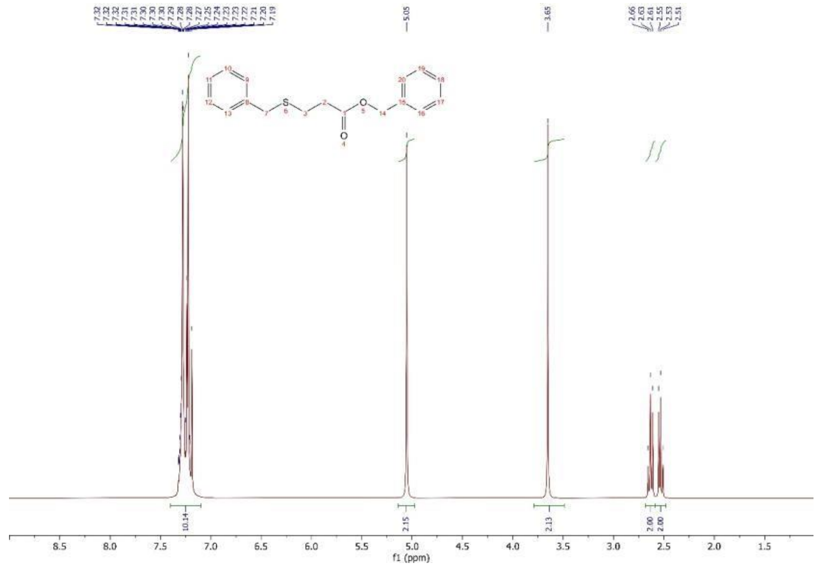

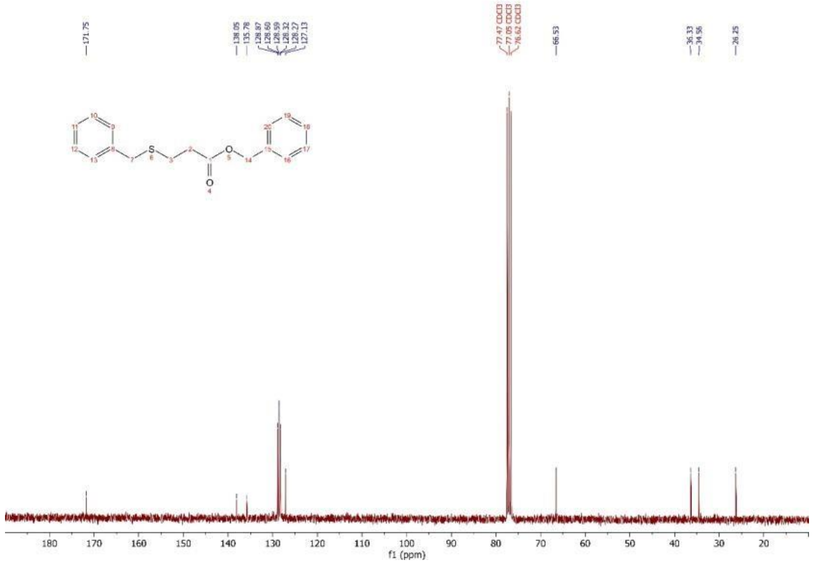
171.75
138.05
135.78
128.87
128.60
128.59
128.32
128.27
127.13
77.47 CDCl3
77.05 CDCl3
76.62 CDCl3
66.53
36.33
34.56
26.25
f1 (ppm)

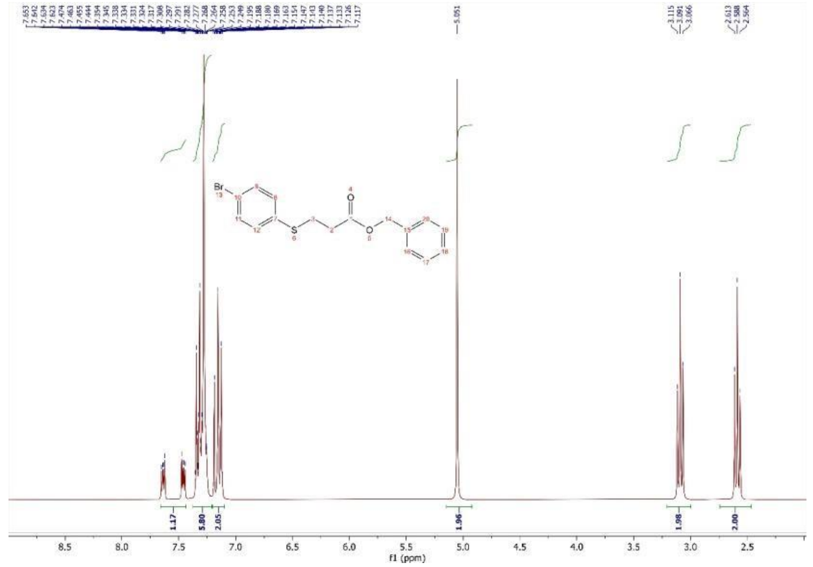
5.051
3.115
3.091
3.066
2.613
2.588
2.564
Br
1.17
5.80
2.05
1.96
1.98
2.00
f1 (ppm)

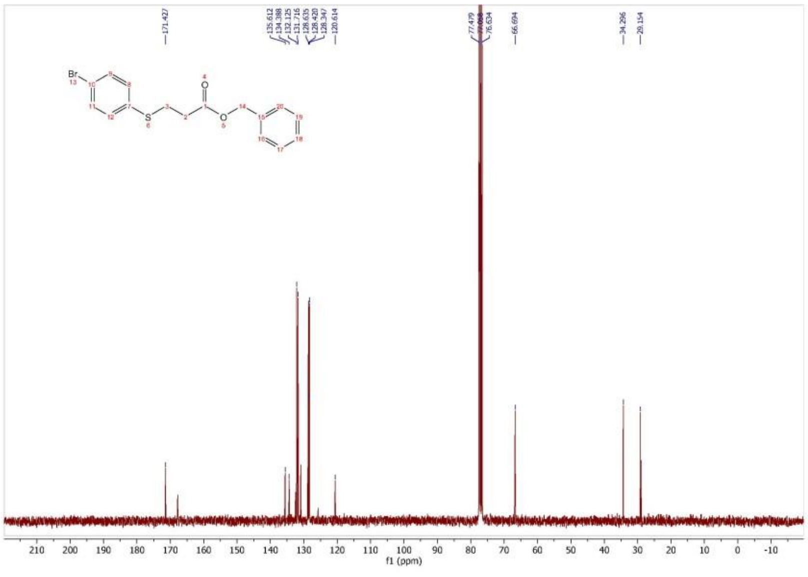

f1 (ppm)

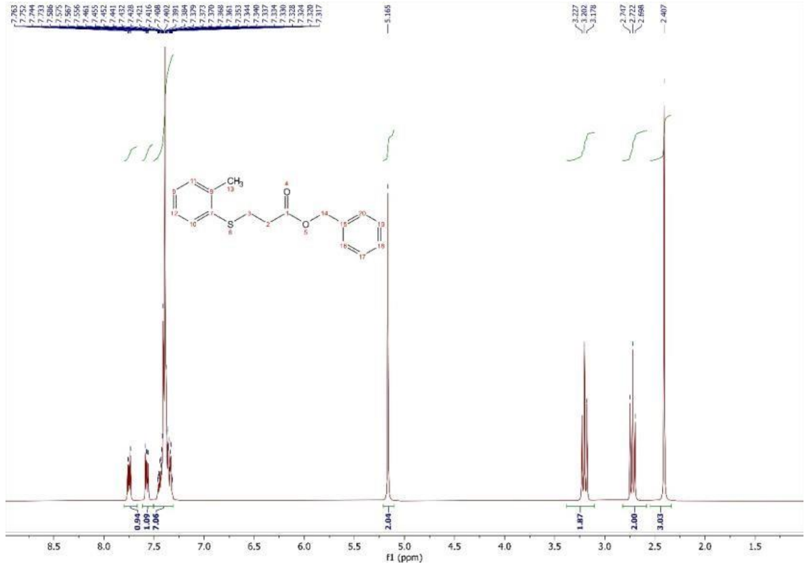

f1 (ppm)

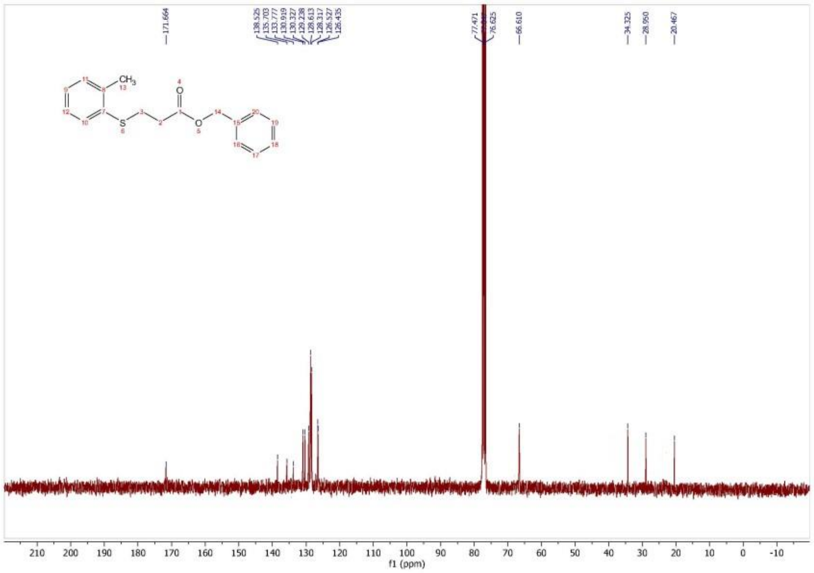
171.664
138.525
135.703
133.777
130.919
130.327
129.238
128.613
128.317
126.527
126.435
77.471
76.625
66.610
34.325
28.950
20.467
CH3
S
O
f1 (ppm)

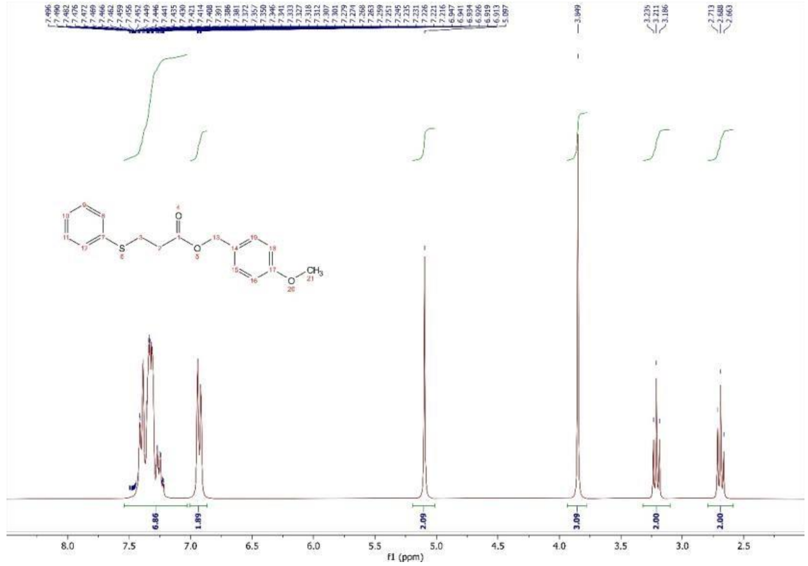
3.849
3.235
3.211
3.186
2.713
2.688
2.663
5.097
6.86
1.89
2.09
3.09
2.00
2.00
S
O
CH3
f1 (ppm)

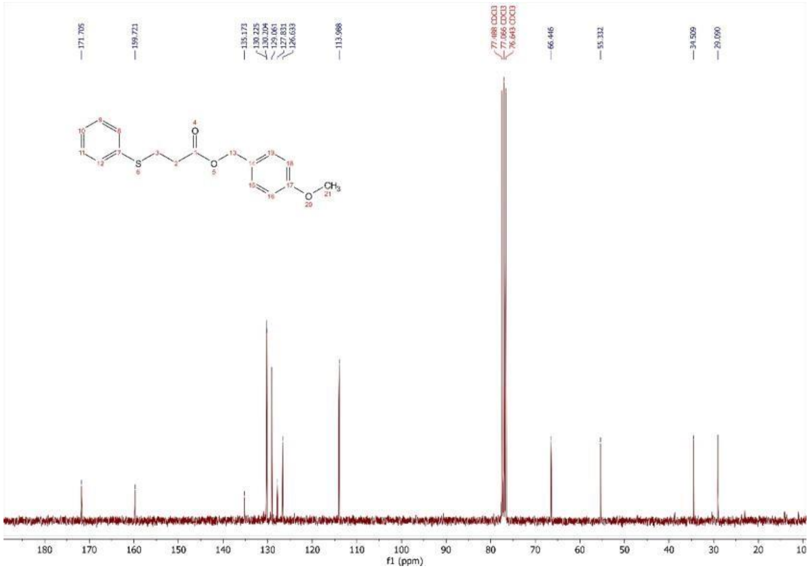
171.705
159.721
135.173
130.225
130.204
129.061
127.831
126.633
113.988
77.488 CDCl3
77.066 CDCl3
76.643 CDCl3
66.446
55.332
34.509
29.090
f1 (ppm)

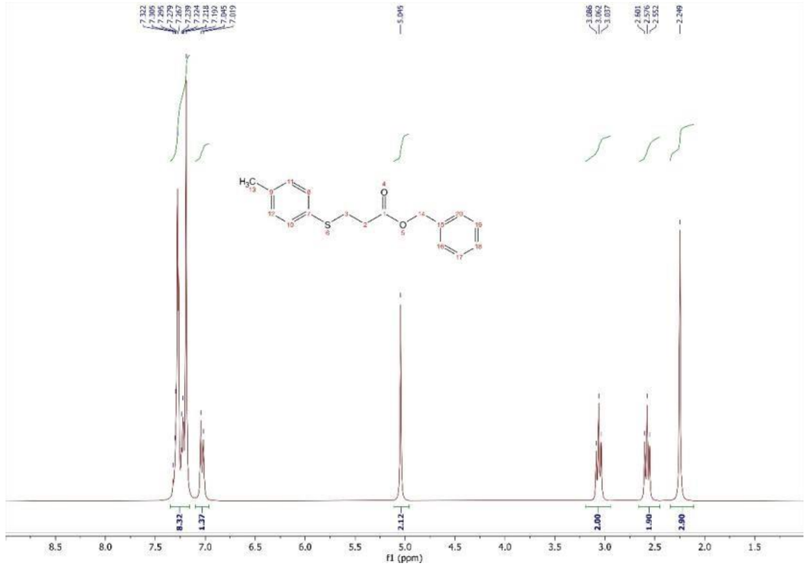
5.045
3.086
3.062
3.037
2.601
2.576
2.552
2.249
8.32
1.37
2.12
2.00
1.90
2.00
f1 (ppm)

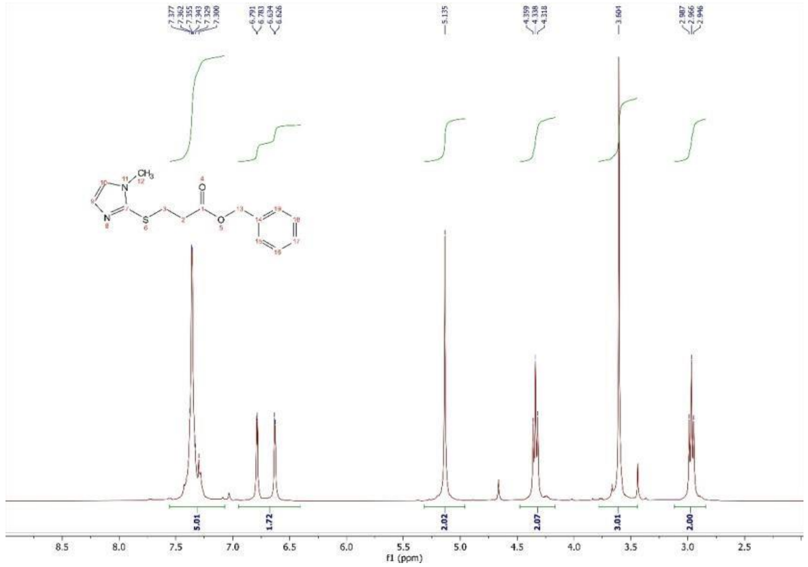
7.377
7.362
7.355
7.343
7.329
7.300
6.791
6.783
6.634
6.626
5.135
4.359
4.338
4.318
3.604
2.987
2.966
2.946
5.01
1.72
2.02
2.07
3.01
2.00
f1 (ppm)

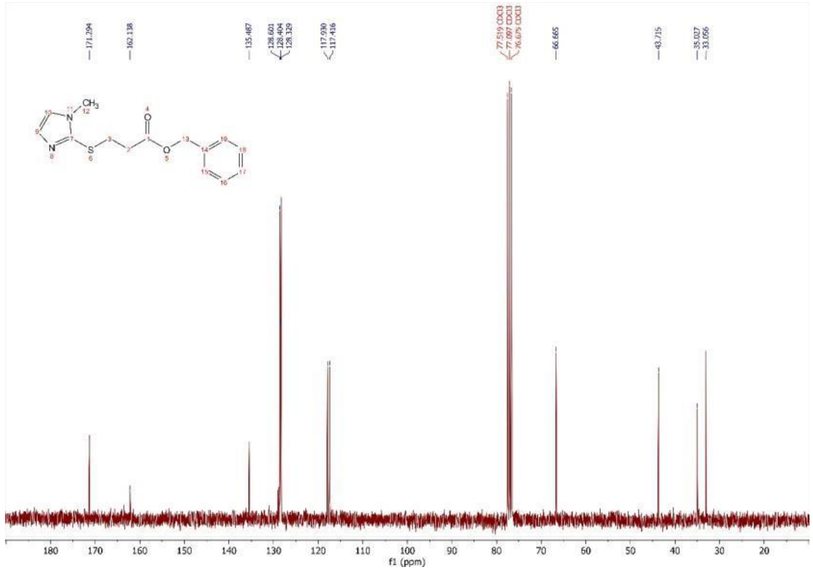
171.294
162.138
135.487
128.601
128.404
128.329
117.930
117.416
77.519 CDCl3
77.097 CDCl3
76.675 CDCl3
66.665
43.715
35.027
33.056
f1 (ppm)

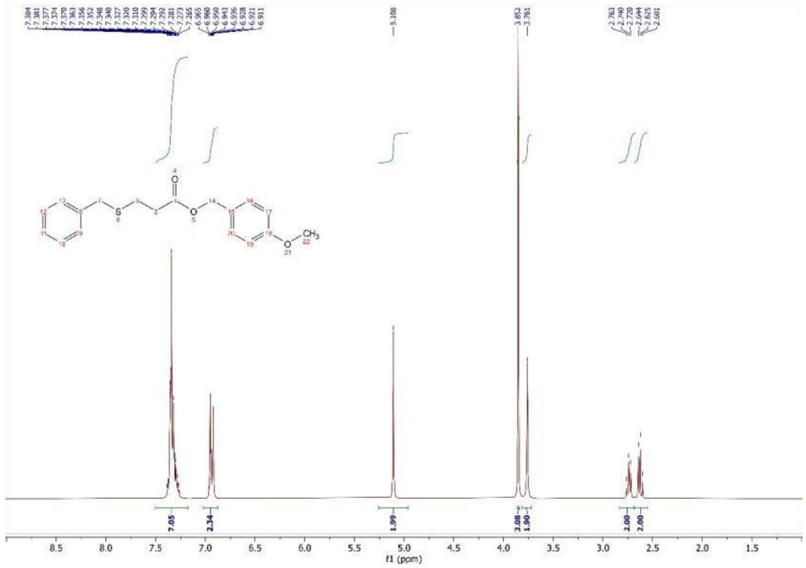

5.108
3.852
3.761
7.05
2.34
1.99
3.08
1.90
2.00
2.00
f1 (ppm)

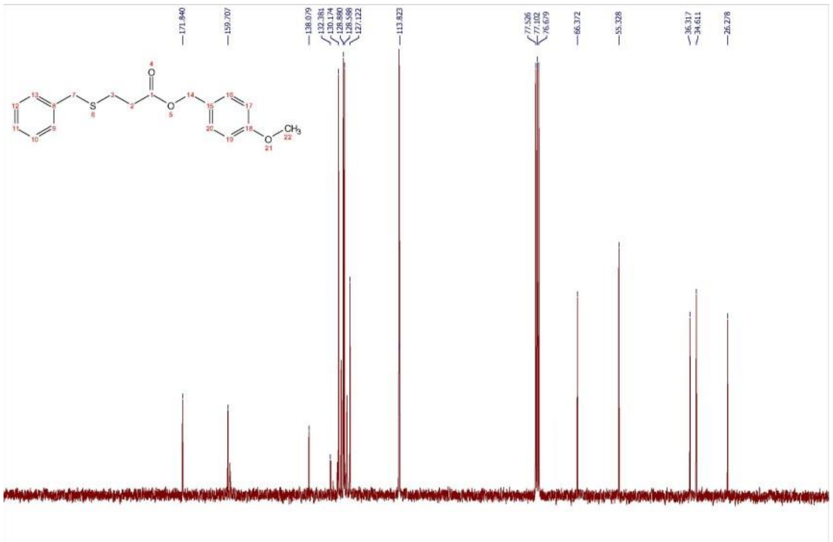

171.840
159.707
138.079
113.823
66.372
55.328
36.317
34.611
26.278
f1 (ppm)

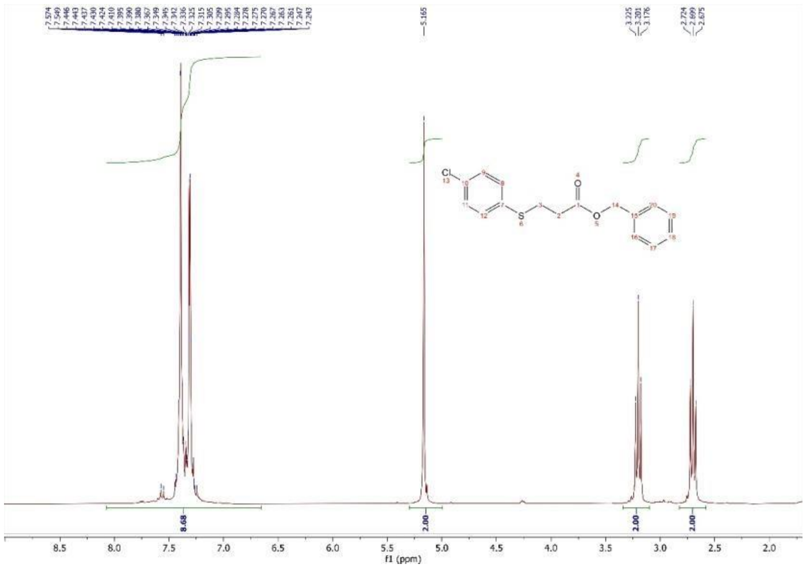

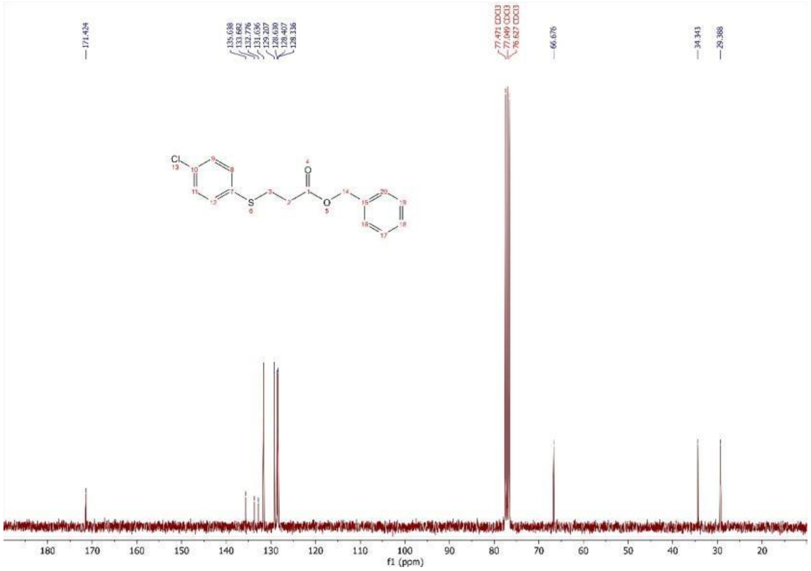

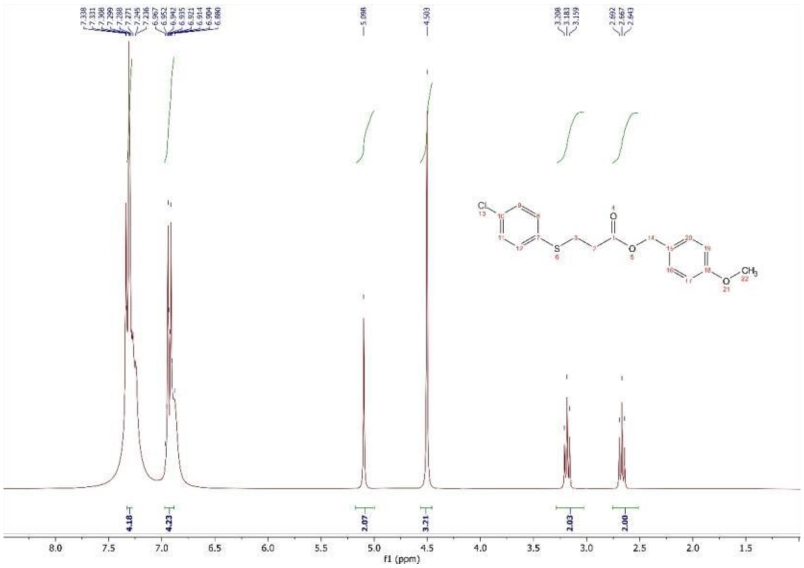

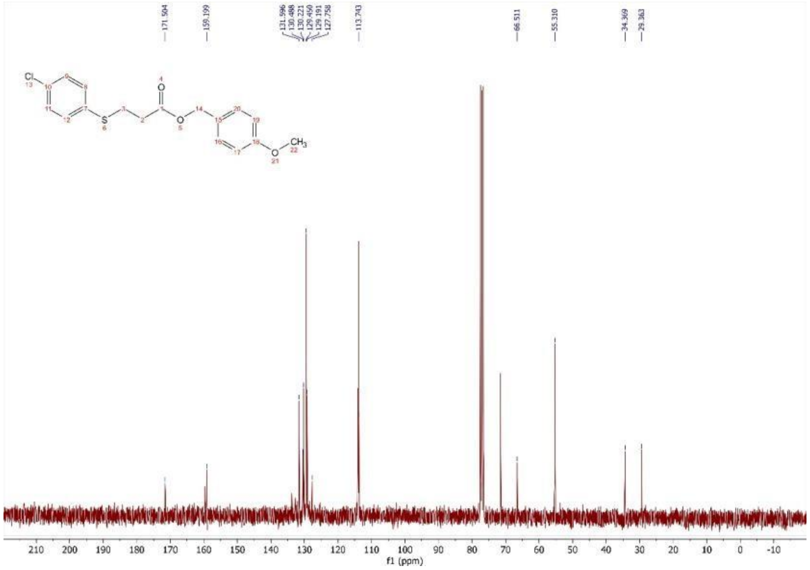

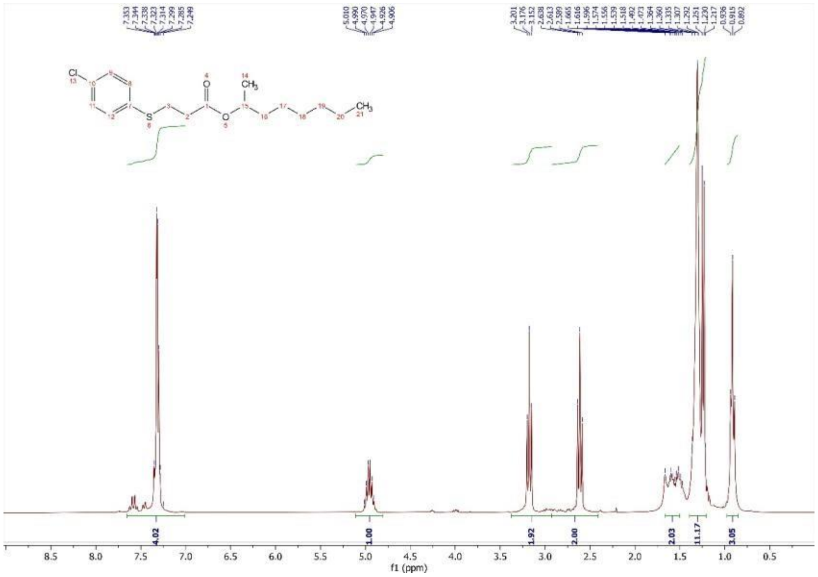
4.02
1.00
1.92
2.00
2.03
11.17
3.05
f1 (ppm)

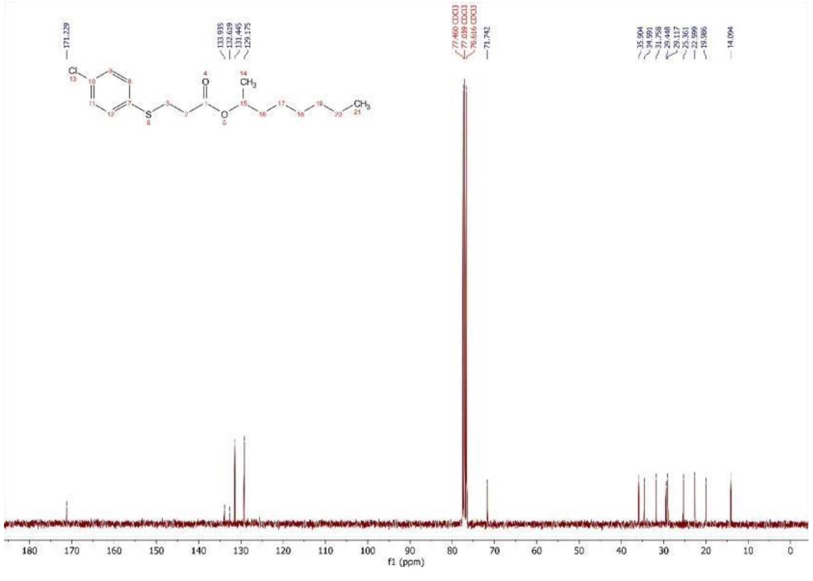
171.229
133.935
132.619
131.445
129.175
77.460 CDCl3
77.038 CDCl3
76.616 CDCl3
71.742
35.904
34.991
31.758
29.448
29.117
25.361
22.599
19.986
14.094
f1 (ppm)

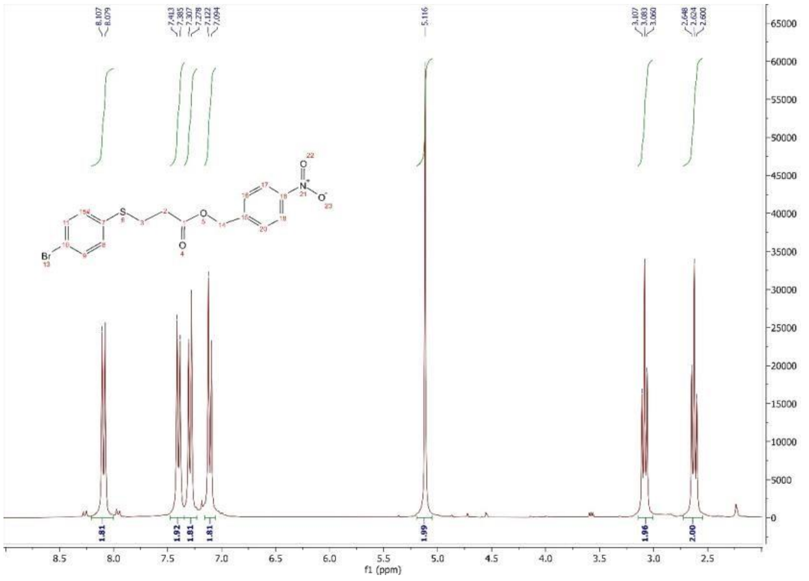

8.107
8.079
7.413
7.385
7.307
7.278
7.122
7.094
5.116
3.107
3.083
3.060
2.648
2.624
2.600
Br
1.81
1.92
1.81
1.81
1.99
1.96
2.00
f1 (ppm)

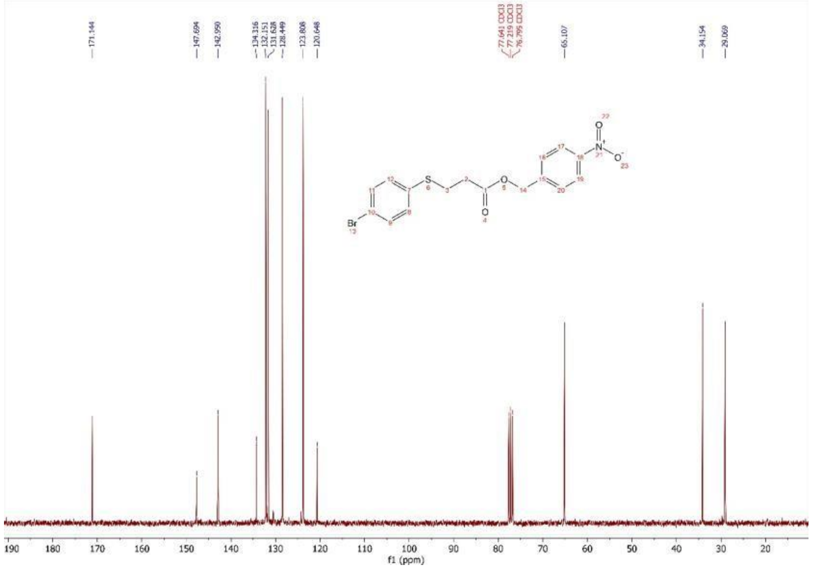

171.144
147.694
142.950
134.316
132.151
131.628
128.449
123.808
120.648
77.641 CDCl3
77.219 CDCl3
76.795 CDCl3
65.107
34.154
29.069
Br
f1 (ppm)

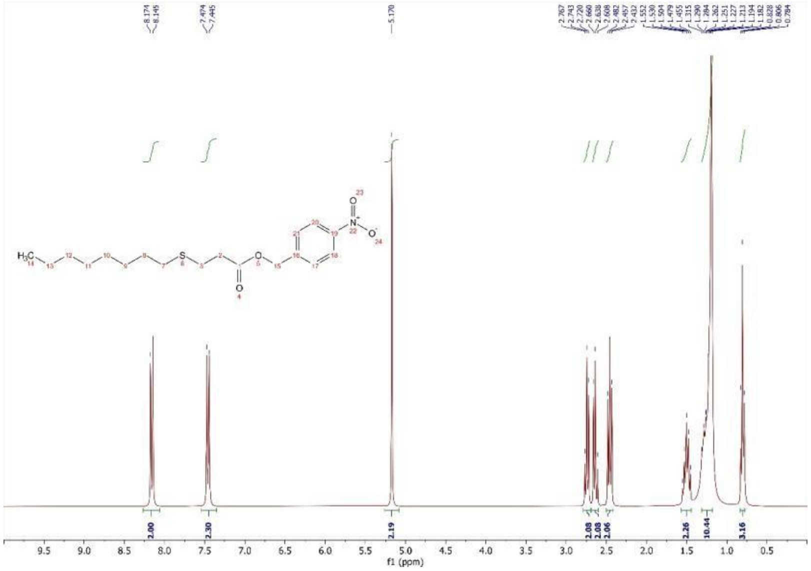

8.174
8.145
7.474
7.445
5.170
2.00
2.30
2.19
2.08
2.08
2.06
2.26
10.44
3.16
f1 (ppm)

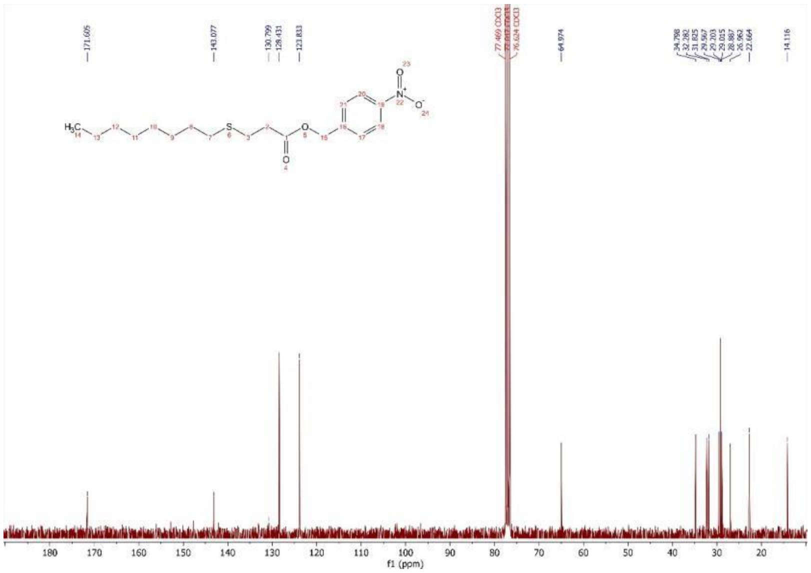

171.605
143.077
130.799
128.431
123.833
77.469 CDCl3
76.624 CDCl3
64.974
34.798
32.282
22.664
14.116
f1 (ppm)

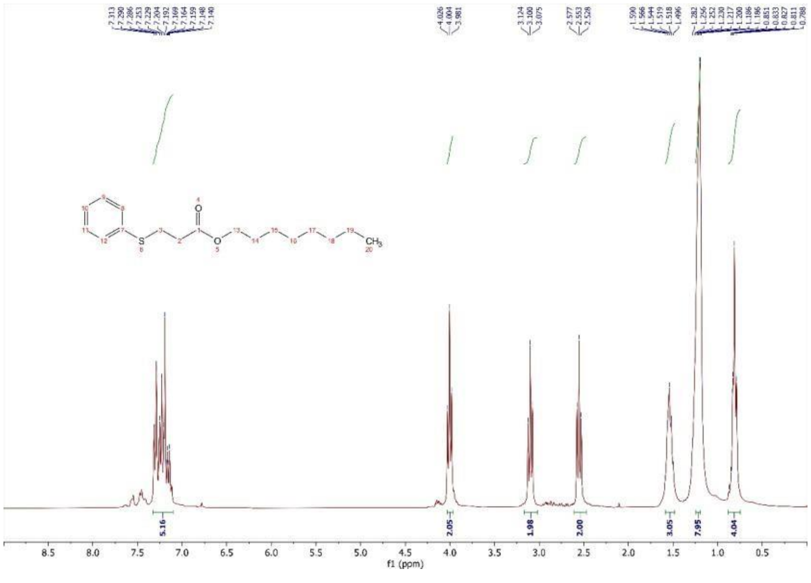

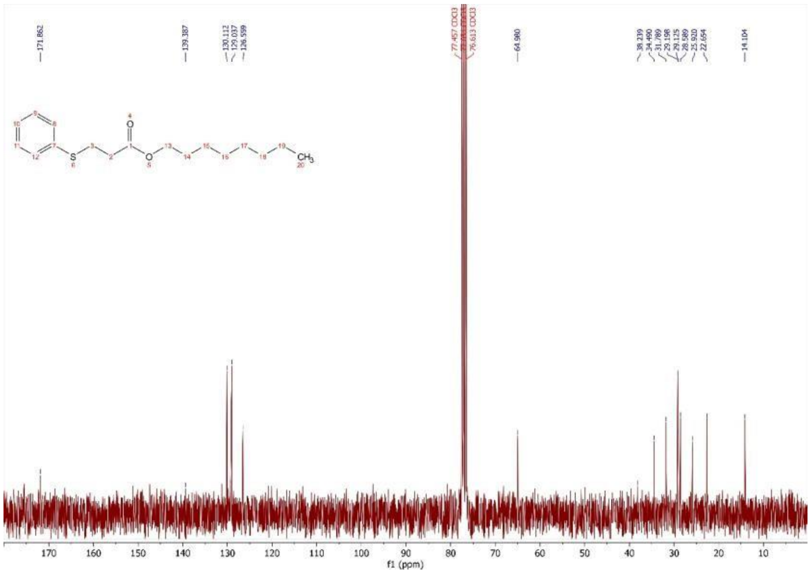

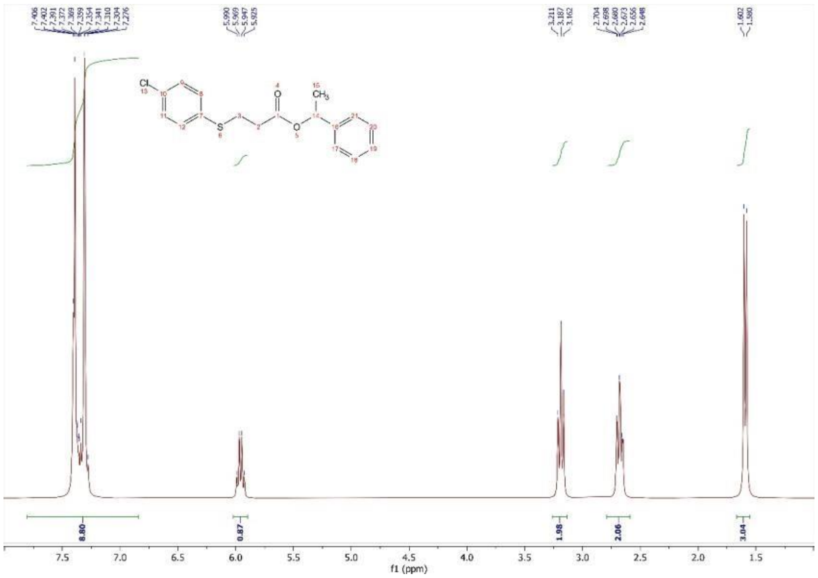

7.406
7.402
7.391
7.372
7.369
7.359
7.354
7.341
7.310
7.304
7.296
5.990
5.969
5.947
5.925
3.211
3.187
3.162
2.704
2.698
2.680
2.673
2.656
2.648
1.602
1.580
8.80
0.87
1.98
2.06
3.04
f1 (ppm)

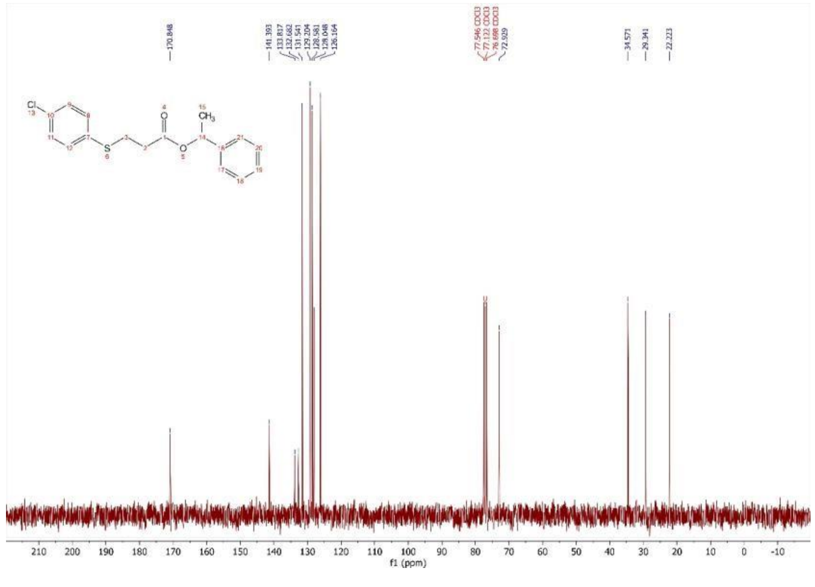

170.848
141.393
133.817
132.682
131.541
129.204
128.581
128.048
126.164
77.546 CDCl3
77.122 CDCl3
76.698 CDCl3
72.929
34.571
29.341
22.223
f1 (ppm)
